# Immunoengineering of a Photocaged 5´‐triphosphate Oligoribonucleotide Ligand for Spatiotemporal Control of RIG‐I Activation in Cancer

**DOI:** 10.1002/anie.202423321

**Published:** 2025-04-21

**Authors:** Sandra Anika Lewash, Vivien Rose McKenney, Christine Wuebben, Janos Ludwig, Racha Hosni, Dirk Radzey, Marieta I. Toma, Eva Bartok, Martin Schlee, Thomas Zillinger, Alexander Heckel, Gunther Hartmann

**Affiliations:** ^1^ Institute of Clinical Chemistry and Clinical Pharmacology – Immunology in Translation University Hospital Bonn Venusberg‐Campus 1 53127 Bonn Germany; ^2^ Institute for Organic Chemistry and Chemical Biology Goethe University Frankfurt Max‐von‐Laue‐Straße 9 60438 Frankfurt am Main Germany; ^3^ Institute of Pathology University Hospital Bonn Venusberg‐Campus 1 53127 Bonn Germany; ^4^ Institute of Experimental Haematology and Transfusion Medicine University Hospital Bonn Venusberg‐Campus 1 53127 Bonn Germany; ^5^ Department of Biomedicine Aarhus University Høegh‐Guldbergs Gade 10 Aarhus 8000 Denmark

**Keywords:** Cancer immunotherapy, Innate immunity, Photocage, RIG‐I, Therapeutic oligonucleotide

## Abstract

Photochemical control of oligonucleotides bears great potential for the spatio‐temporal control of therapeutic targets, such as immune sensing receptors. Retinoic acid‐inducible gene I (RIG‐I) is a cytoplasmic receptor of the innate immune system that triggers antiviral responses upon detection of viral RNA. RIG‐I can be specifically activated by short double‐stranded (ds) RNA with a blunt 5′ end bearing a triphosphate, mimicking nascent viral transcripts. Tumor cells are specifically sensitive to RIG‐I‐induced cell death. Here we developed a potent oligonucleotide ligand for spatiotemporally controlled activation of RIG‐I by light exposure. Through structural considerations and functional studies we identified a combination of two nucleoside positions in a RIG‐I oligonucleotide ligand for which the substitution of both respective 2′‐hydroxy groups of the ribose by photolabile protecting groups (2′‐photocages) resulted in a complete loss of RIG‐I ligand activity, whereas photocaging the individual positions was not sufficient to turn off RIG‐I. Light exposure fully restored RIG‐I activation by the photocaged RIG‐I ligand, enabling light‐controlled RIG‐I‐mediated cell death of human cancer cells which had internalized the photocaged RIG‐I ligand prior to light exposure. This novel photoactivatable RIG‐I oligonucleotide ligand may be applicable for precise light‐controlled induction of tumor cell death in superficial cancer such as melanoma.

## Graphical abstract

This study presents a method for precise control of RIG‐I (retinoic acid inducible gene I), an innate immune receptor of RNA that, when activated, induces anti‐tumor immune responses and cancer cell death. By incorporating photocage modifications into RNA ligands, we enable a spatially and temporally regulated immune response and offer a targeted strategy for cancer therapy while minimizing systemic effects.

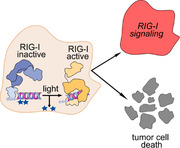

## Introduction

In recent years, light has become a popular tool for the selective control of biochemical processes.^[^ ^1^ ^]^ It provides an ideal external, non‐invasive trigger that can allow the exact spatial and temporal activation or deactivation of functional molecules. In particular, photopharmacology has become a major driver of innovation by enabling the precise on‐target effectivity of pharmacological therapies.^[^ ^2^, ^3^ ^]^ For example, Trauner et al. demonstrated how tadpoles can be reversibly anesthetized with light of two different wavelengths.^[^ ^4^ ^]^ Much progress has been made in the development of irreversible photo‐triggering or reversible photoswitching technologies.^[^ ^5^ ^]^

Therapeutic oligonucleotides are a promising new field of application for photopharmacological tools. In 2023, four oligonucleotides were approved by the Federal Drug Administration (FDA).^[^ ^6^ ^]^ Altogether, more than 20 oligonucleotide‐based therapies are now available, including aptamers, antisense oligonucleotides (ASO) DNA, and small interfering RNA (siRNA), and immunostimulatory oligonucleotides as immune adjuvants in vaccines (e.g., CpG 7909 in CYFENDUS, License No. 2089, July 2023).^[^ ^7^, ^8^ ^]^ Chemical modification of oligonucleotides is essential for achieving the desired biological properties such as increasing nuclease stability or avoidance of immune recognition by the innate immune system, thereby reducing unwanted inflammatory side effects. In general, modifications of nucleic acids range from backbone alterations and base modifications to branched RNA hybrids and the incorporation of photolabile protecting groups as well as photoswitches.^[^ ^9^, ^10^, ^11^, ^12^, ^13^, ^14^, ^15^, ^16^ ^]^ Photolabile protecting groups represent a particularly useful approach. By enabling light‐controlled activation of oligonucleotides, photolabile protecting groups offer precise spatial and temporal control of functional activities such as protein binding of aptamers or Watson–Crick base pairing to target nucleic acid by ASO DNA and siRNA.

Immunostimulatory oligonucleotides, unlike ASO and siRNA, are not based on Watson–Crick pairing to a target molecule but instead mimic specific, molecular characteristics of pathogen‐derived DNA or RNA. These oligonucleotides activate the nucleic acid receptors of the innate immune system, thus inducing direct anti‐microbial and anti‐tumor responses as well as initiating and forming the subsequent adaptive immune response. Immunostimulatory oligonucleotides can potentially be applied as vaccine adjuvants, prophylactic and therapeutic antivirals and for tumor immunotherapy.

Retinoic acid‐inducible gene (RIG‐I) is a key innate immune RNA receptor, expressed in the cytoplasm of all nucleated cells, where it acts as an essential sensor of RNA viruses. Upon activation, RIG‐I induces type I interferons (IFN), pro‐inflammatory cytokines, and antiviral effector proteins.^[^ ^17^, ^18^, ^19^ ^]^ Potent RIG‐I activation can also induce inflammatory cell death, particularly in tumor cells, with classical “immunogenic” hallmarks such as HMGB1 (high mobility group box 1) release and calreticulin exposure on the cell surface.^[^ ^20^, ^21^, ^22^ ^]^ Intratumoral activation of RIG‐I exhibits features of a cancer vaccine by simultaneously inducing the release of tumor antigens and creating a pro‐immunogenic environment that facilitates the development of tumor‐specific cytotoxic T cells.^[^ ^20^, ^21^ ^]^ Specific agonists for RIG‐I have been developed for applications both in tumor immunotherapy and the prophylaxis and treatment of viral infections.^[^ ^23^, ^24^, ^25^, ^26^, ^27^, ^28^, ^29^, ^30^ ^]^

**Figure 1:**
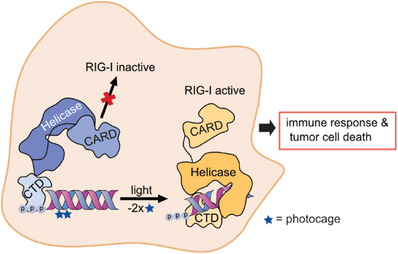
Overview of the concept of light‐induced control of RIG‐I activation. The C‐terminal Domain (CTD) of RIG‐I binds ppp‐dsRNA. In a second step, the CTD bound ppp‐dsRNA displaces CARD (caspase activation and recruitment domain) from inhibitory helicase binding leading to the initiation of downstream signaling and type I IFN induction. Photocage RIG‐I ligand: In the absence of suitable light, helicase (E510) interaction with bound ppp‐dsRNA is sterically blocked by photocages. Upon irradiation with light, the photocages are cleaved from the ligand enabling CARD displacement and RIG‐I signaling.

Anti‐tumor therapies are inherently cytotoxic, and the inability to confine therapeutic effects to the tumor area can lead to severe adverse effects, such as systemic inflammation.^[^ ^31^, ^32^ ^]^ Thus, spatiotemporal control of RIG‐I activating oligonucleotides to allow selective activation in the tumor site would be highly beneficial, both in limiting side effects and allowing for higher, local oligonucleotide doses. In this study, we investigated how to achieve photochemical control of RIG‐I ligand activity by adding photolabile protecting groups at different positions of a RIG‐I‐activating oligonucleotide. Based on previous insight into the structure of RIG‐I ligands and functional testing of several candidate positions,^[^ ^33^ ^]^ we were able to identify two, specific 2′‐hydroxy sites which in combination allowed for a robust light‐dependent spatiotemporal control of RIG‐I ligand activity. This designed RIG‐I ligand demonstrated stable off‐properties in cellulo, and fully turned‐on RIG‐I activity upon exposure of cells to light, enabling controlled cell death induction in human primary tumor cells (Figure 1).

## Results and Discussion

### Site‐Specific 2′‐*O*‐Methyl Screening Identifies Candidate Sites for 2′‐*O*‐Photocage Modification

**Figure 2:**
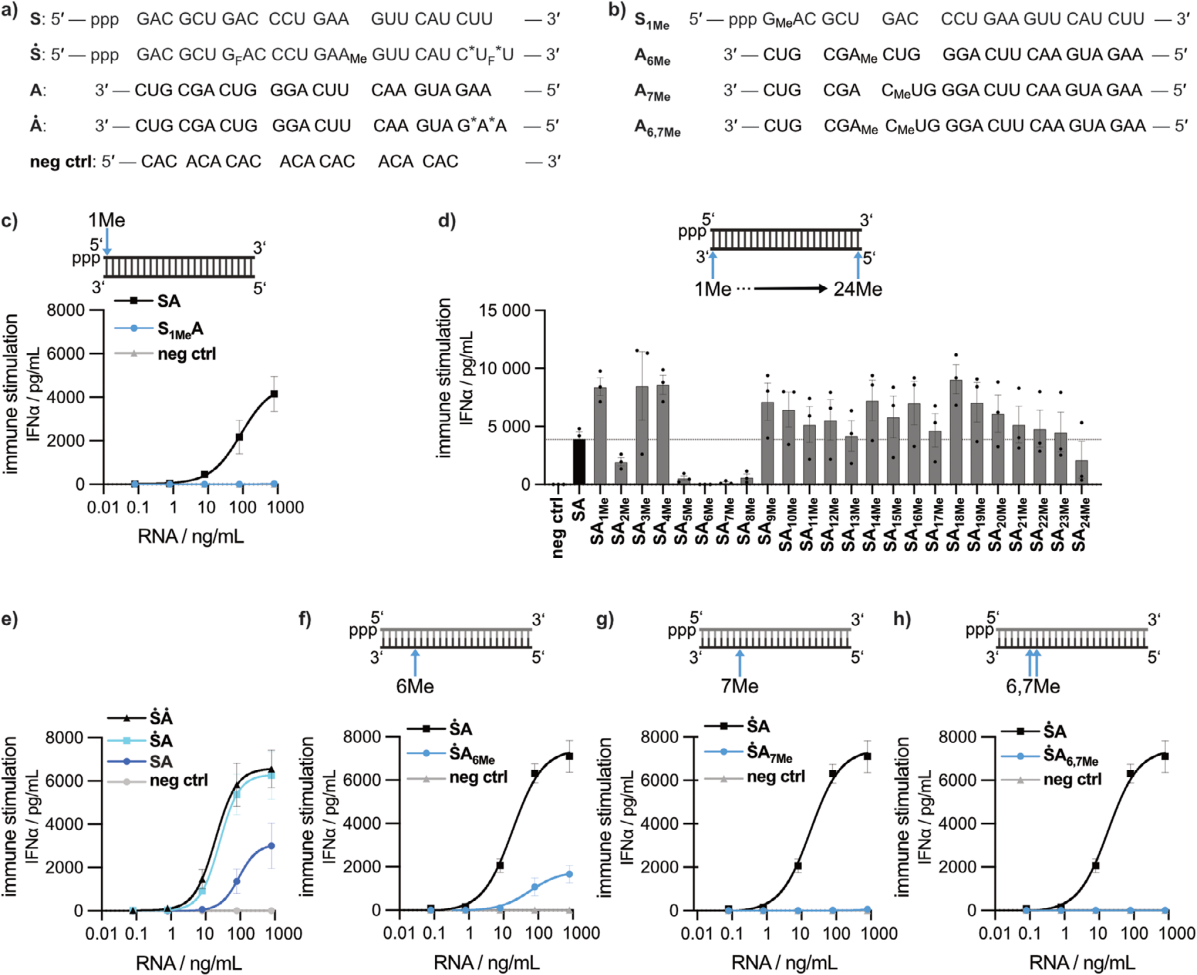
2′‐*O*‐Methyl‐modification screening of ppp‐dsRNA ligands for RIG‐I activation in PBMC, as measured by the antiviral protein IFN‐alpha, identifies candidate nucleotide positions for the introduction of 2′‐photocage modifications. a) Sequences of single‐stranded RNA oligonucleotides (**S**ense: S; **A**ntisense: A) used for the formation of double‐stranded RNA RIG‐I ligands (**SA/ṠA/ṠȦ**), as well as the sequence of the single‐stranded negative control RNA (**neg ctrl**). b) Example sequences for 2′‐*O*‐methyl‐modified RNA strands. c) Peripheral blood mononuclear cells (PBMC) were transfected with the indicated concentrations of **SA**, **S1MeA** or negative control RNA. d) To screen for suitable 2´‐*O*‐photocage positions in the antisense strand, PBMC were transfected with 800 ng mL^−1^ of the indicated double‐stranded RNA or negative control RNA. e)–h) PBMC were transfected with oligonucleotides as indicated. 24 h after transfection of oligonucleotides, IFN‐α was measured in the supernatants by ELISA. Data are shown as the means ± SEM of at least three independent experiments. ppp: triphosphate, F: 2′‐deoxy‐2′‐fluoro, Me: 2′‐*O*‐methylation, *: phosphorothioate.

Based on our previous studies on RIG‐I‐RNA binding and activity, we hypothesized that 2′‐*O*‐photocage modifications of certain nucleotide positions would block RIG‐I activation through steric hindrance. To identify such sites, we screened 2′‐*O*‐methylations of a prototypical RIG‐I oligonucleotide ligand (double‐stranded RNA termed **SA**, S for **S**ense and A for **A**ntisense, Figure 2a). As expected from previous findings,^[^ ^33^ ^]^ the addition of a 2′‐*O*‐methyl group at the first nucleotide of the sense strand (→**S_1Me_**, Figure 2b) completely inhibited the release of the immune signaling molecule IFN‐alpha, a type I interferon that represents a biomarker for viral infection and at the same time functions as antiviral effector protein, from human peripheral blood mononuclear cells (PBMC) (**S_1Me_A**, Figure 2c). Furthermore, in published crystal structures of RIG‐I bound to dsRNA,^[^ ^34^, ^35^ ^]^ the amino acids Q507, E510, and Q511 in the Hel2i domain of RIG‐I interact with the 2′‐hydroxy group of the nucleoside positions that correspond to **S_6_**, **A_7_**, and **S_8_** of our dsRNA ligand **SA**. To assess the relative contribution of the individual nucleotide positions of the antisense strand to RIG‐I activity, we 2′‐*O*‐methylated each of the 24 nucleotide positions of the antisense strand. Of note, the positions of the antisense strand were counted from the 3′‐end, while positions of the sense strand (carrying the 5´‐PPP) were counted from the 5′‐end (Figure 2a). While at most of the positions, 2′‐*O*‐methylation had no effect or even enhanced RIG‐I stimulation, four positions inhibited by over 90%, **SA_5Me_, SA_6Me_, SA_7Me_, SA_8Me_**, with positions **SA_6Me_** and **SA_7Me_** reducing activity below the detection limit of the assay (Figure 2d).

Next, phosphorothioate‐, 2′‐*O*‐methyl‐ and 2′‐deoxy‐2′‐fluoro‐modifications were introduced into the sense strand in order to protect RNA from degradation by RNases, which are abundant in biological environments. The positions chosen for these modifications are based on previous screenings in our lab (**Ṡ**, Figure 2a).^[^ ^36^ ^]^ Accordingly, **ṠA** exhibited higher potency than **SA** (Figure 2e). **Ṡ** in combination with **A_6Me_** (**ṠA_6Me_**) showed some remaining RIG‐I activity (Figure 2f), whereas for the combination of **Ṡ** with **A_7Me_** (**ṠA_7Me_**) RIG‐I activity was below the detection limit (Figure 2g). The combination of the two (**ṠA_6,7Me_**) again abolished RIG‐I activity. From these data we concluded that the combination of the two modifications with the highest inhibitory potential (**A_6,7Me_**) may add a favorable safety margin for photocaging the RIG‐I oligonucleotide ligand.

Moreover, since terminal phosphorothioates block exonuclease‐mediated degradation of oligonucleotides,^[^ ^37^ ^]^ two additional phosphorothioates were added to the 5′‐end of the antisense strand in all subsequent designs of photocaged ligands. In addition, a monophosphate was added to the 5′‐end of the antisense strand to prevent the binding of RIG‐I at this end (→**Ȧ**, Figure 2a,e).^[^ ^38^ ^]^

### Synthesis of 2′‐*O*‐Photocage Modified Oligonucleotides

**Scheme 1:**
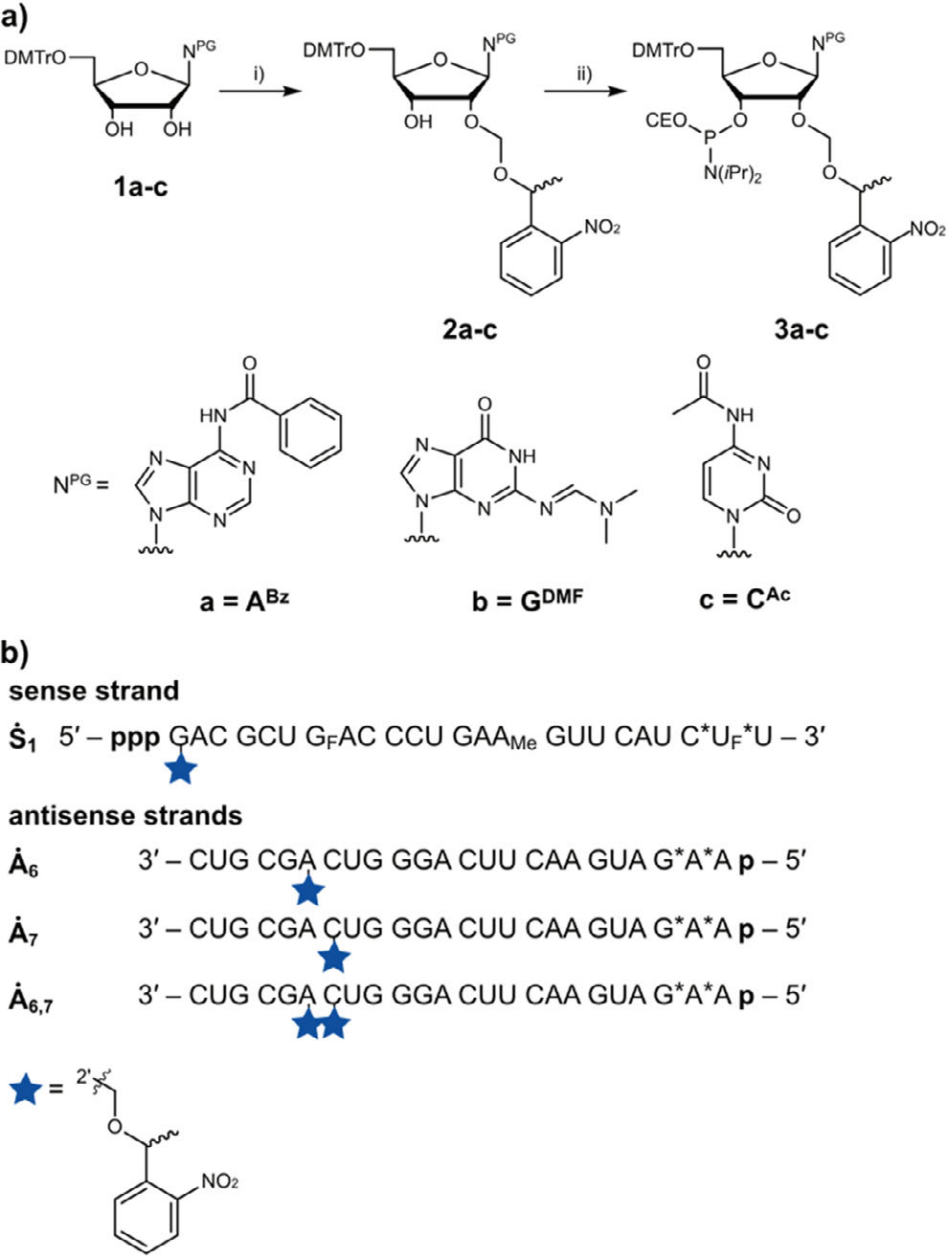
Synthesis of photolabile protected phosphoramidites and RNA ligands. a) i) Bu_2_SnCl_2_, DIPEA, ClCH_2_CH_2_Cl, rt, 1 h, *R,S*‐1‐(1‐(chloromethoxy) ethyl)‐2‐nitrobenzene, 80 °C, 30 min, 33%. ii) PN(*i*Pr)_2_OCE‐Cl, MeCN, microwave, 40 °C, 1 h, 45%. b) synthesized light‐activatable oligonucleotides **Ṡ_1_**, **Ȧ_6_**, **Ȧ_7_**, **Ȧ_6,7_**. p: phosphate, ppp: triphosphate F: 2′‐deoxy‐2′‐fluoro, Me: 2′‐*O*‐methylation, *: phosphorothioate.

Based on the 2′‐*O*‐methylation screen, we introduced photolabile protecting groups at the 2′‐*O*‐positions of the first nucleotide of the sense strand (→**Ṡ_1_**, Scheme 1) and the 6th and the 7th nucleotide of the antisense strand (→**Ȧ_6_**, **Ȧ_7_** and **Ȧ_6,7_**). NPE (*1‐(2‐nitrophenyl) ethyl*) was selected as the photolabile group. For steric reasons, we inserted a formaldehyde acetal between the 2′‐hydroxy group and the NPE photocage according to Pitsch et al.^[^ ^39^ ^]^ To obtain the required phosphoramidites, we initially installed the caging group on protected nucleoside precursors **1a‐c** (→**2a‐c**, see Scheme 1a). This was performed by temporary protection of the 2′‐ and 3′‐hydroxy groups with Bu_2_SnCl_2_ and regioselective opening. Finally, phosphitylation yielded the amidites **3a‐c**. Standard procedures during the solid‐phase synthesis, cleavage from the solid‐support as well as deprotection of the nucleobases were used to obtain the caged, modified oligonucleotides shown in Scheme 1b. For the photocaged sense strand **Ṡ_1_**, an additional triphosphorylation step after the solid‐phase synthesis was necessary.^[^ ^40^ ^]^

### Control of RIG‐I Activity by 2′‐*O*‐Photocage Modified Ligands

It should be noted that upon the cleavage of the photocage by light, a 2′‐hydroxy group remains at its position.^[^ ^38^ ^]^ Thus, after irradiation, the photocaged ligand presents the same structure as the primarily uncaged RNA (e.g., **ṠȦ**). The presence of the 2′‐hydroxy groups can also be deduced from the LC‐MS data obtained after cleavage (Table S5). To see whether the synthesized photocaged strands enable control of immune activation, the respective double‐stranded RNA molecules (1.28–800 ng mL^−1^) were transfected into peripheral blood mononuclear cells (PBMC), then irradiated using light emitting diodes (LED) at 365 nm wavelength (2 mW) for 10 min (indicated by + hν), and IFN‐α‐levels were measured 24 h later in cellular supernatants.

**Figure 3:**
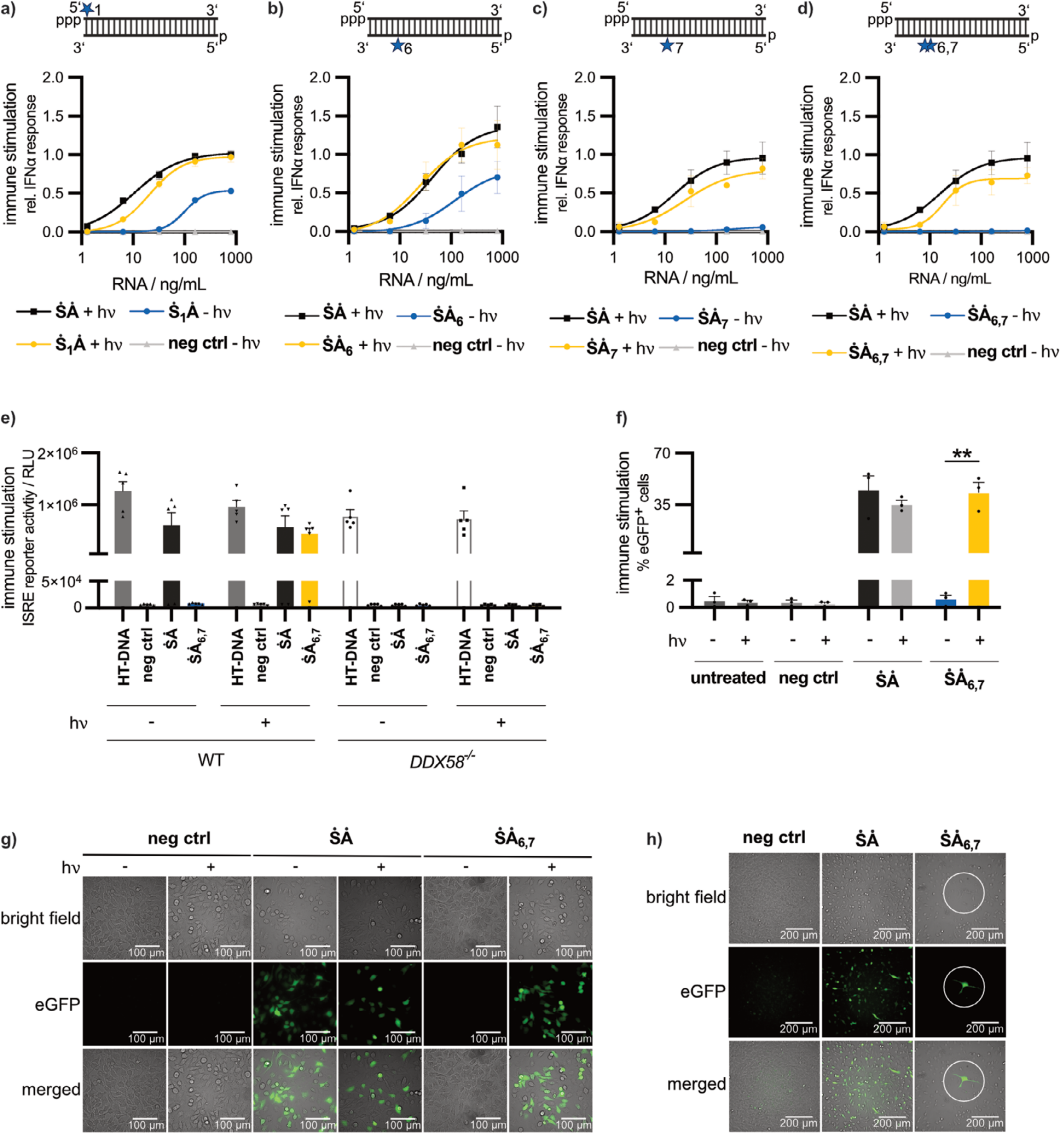
The combination of two 2´‐*O*‐photocages at nucleotide positions 6 and 7 of the antisense strand of the RIG‐I oligonucleotide ligand allows robust light‐mediated control of RIG‐I activation. a–d) PBMC were transfected with oligonucleotides as indicated. IFN‐α was measured in the supernatants (24 h) by ELISA. Results are normalized to **ṠȦ** (800 ng mL^−1^) without light irradiation. Untreated controls and negative control with light irradiation are not depicted (results similar to negative control). e) THP1 dual cells (InvivoGen) wildtype (WT) or RIG‐I knockout (*DDX58^−/−^)* cells were transfected with 800 ng mL^−1^ photocaged **ṠȦ_6,7_** or control RNA. Lucia luciferase readout for the ISRE reporter was performed in the supernatant after 24 h of incubation. Untreated control not depicted (similar to negative control). f–h) A549 IFNβ‐EGFP reporter cells were transfected with 1600 ng mL^−1^ photocaged **ṠȦ_6,7_** or control RNA, imaged 48 h after transfection. f) Percentage of eGFP^+^ cells. Two‐tailed unpaired *t*‐test, *P ≤ 0.05, **P ≤ 0.01. g), h) Images were taken on a fluorescence microscope using bright field (top row) or fluorescence (eGFP, middle row). g) 40× magnification, untreated samples are not shown (similar to negative control in f), images shown are representative of two independent experiments. h) 20× magnification, white circle represents area of irradiation (365 nm, 2 mW, 10 min), images shown are representative of two independent experiments. a–f) Data are shown as means ± SEM of three independent experiments. + hν: samples irradiated with light emitting diodes (365 nm, 2 mW, 10 min) after transfection; − hν samples were not irradiated with light.

Unexpectedly, **Ṡ_1_Ȧ** modification did not result in a complete loss of immune activation (Figure 3a). However, using LC‐MS we detected ≤ 2% uncaged product in the **Ṡ_1_** sample (Figure S1), which could not be eliminated by repeated purification. Thus, the small amount of uncaged product likely contributes to the remaining activity of **Ṡ_1_Ȧ**. The same observation was made with either **Ȧ_6_** or **Ȧ_7_** (Figures S3, S5), with both **ṠȦ_6_** and **ṠȦ_7_** exhibiting residual activity in the absence of light (Figure 3b,c). For the caged, modified oligonucleotide **Ȧ_6,7_** we found that approximately 5% of RNA is either uncaged at the 6th or the 7th position. However, completely uncaged **Ȧ_6,7_** was never observed in the absence of light (Figure S7). Consistent with the LC‐MS data, the photocaged double‐stranded RIG‐I ligand **ṠȦ_6,7_** demonstrated excellent control of light‐induced activation (Figure 3d) with no detectable activity before irradiation and gain of activity after irradiation. Ten min of irradiation at 2 mW was sufficient to uncage 100% of **ṠȦ_6,7_** at least one position and >80% of **ṠȦ_6,7_** at both positions (Figure S8).

To confirm that 10 min of irradiation did not compromise cell viability and the type I interferon response, we decided to test different irradiation times on cultured cells (Figure S11). Since PBMCs are a heterogenous mixture of cell types and contain short‐lived cell types making them not suitable to investigate subtle differences in cell viability, we used the monocytic THP1 dual cell line. These cells have an integrated type I IFN reporter, consisting of an ISG54 promoter coupled to five interferon‐sensitive response elements (ISRE). This promoter controls the expression of a Lucia luciferase gene, allowing for highly sensitive quantification of the type I IFN response. In THP‐1 dual cells transfected with **ṠȦ**, 10 min of irradiation was well tolerated and did not compromise the type I IFN response or cell viability. Longer irradiation times of 15 and 30 min did not lead to measurable cell death (Figure S11b) but to a slight reduction in the type I IFN response (Figure S11a). Similar results were obtained in the human cervical cancer cell line HeLa with CXCL10 (C‐X‐C motif chemokine 10) production and cell death as readouts (Figure S11c,d). As type I interferon itself can be weak and difficult to measure in many non‐immune and tumor cells, the more sensitive CXCL10 response, which is induced by RIG‐I activation as well as type I IFN receptor signaling, was used as a readout for RIG‐I activation.^[^ ^41^ ^]^ It is not clear if the observed reduction in type I IFN and CXCL10 response at high irradiation times is due to a reduction in cell viability below the threshold of full cell death induction, or another effect of 365 nm light on the cell culture system. Nevertheless, at 10 min irradiation no adverse effects on cells were observed and thus, this condition was maintained in subsequent experiments.

To test whether the immunostimulatory activity observed is RIG‐I‐specific, we tested **ṠȦ_6,7_** in wildtype (WT) or RIG‐I knockout (DDX58−/−) THP1 dual cells (Figure 3e). In contrast to WT cells, RIG‐I knockout cells are not expected to respond to a RIG‐I‐specific ligand. In wildtype cells, high activity was seen for **ṠȦ** as well as for photocaged **ṠȦ_6,7_** followed by irradiation. In contrast, RIG‐I knockout cells showed no activation in response to **ṠȦ** or photocaged **ṠȦ_6,7_** followed by irradiation of cells, indicating that the immunostimulatory activity of the respective RIG‐I ligands in THP1 cells was RIG‐I dependent. As expected, both THP1 dual wildtype and RIG‐I knockout cells responded to the cGAS ligand HT‐DNA (herring testes DNA) which was used as a RIG‐I independent positive control. These results demonstrate that the **ṠȦ_6,7_** design allows for specific spatiotemporal control of RIG‐I activation with light.

To provide further evidence for spatiotemporal control of RIG‐I activation, we generated A549 IFN‐β‐eGFP reporter cells. In these cells, CRISPR‐Cas9 was used to introduce an IFN‐β‐promoter with an eGFP (enhanced green fluorescent protein) gene in the IFN‐β‐locus. As a result, the cells express green fluorescence (eGFP) in response to a stimulus that induces the expression of IFN‐β (type I IFN). These A549 IFN‐β‐eGFP reporter cells were transfected with a high dose (1600 ng mL^−1^) of the photocage‐modified **ṠȦ_6,7_**, of **ṠȦ** or of single‐stranded negative control RNA. After transfection, cells were irradiated with light. The percentage of eGFP‐positive cells was quantified by a fluorescence imaging reader (Figure 3f). Cells transfected with the photocaged RIG‐I ligand **ṠȦ_6,7_** exhibited increased numbers of eGFP‐positive cells in response to irradiation. Without irradiation, the fluorescence signal after **ṠȦ_6,7_** transduction was at background levels, similar to the cells treated with negative control RNA with and without irradiation (Figure 3g). We then examined the spatial precision of light‐induced RIG‐I activation. A549 reporter cells were seeded in 6 well plates and transfected with 1600 ng mL^−1^ **ṠȦ_6,7_**, followed by irradiation of a small, circumscribed area (300 µm, white circle, Figure 3h) with light. Resulting IFN‐β‐eGFP fluorescence was only observed in the precise area of irradiation (white circle), demonstrating spatially defined RIG‐I activation.

### Light‐Mediated Spatiotemporal Control of RIG‐I‐Induced Tumor Cell Death

**Figure 4:**
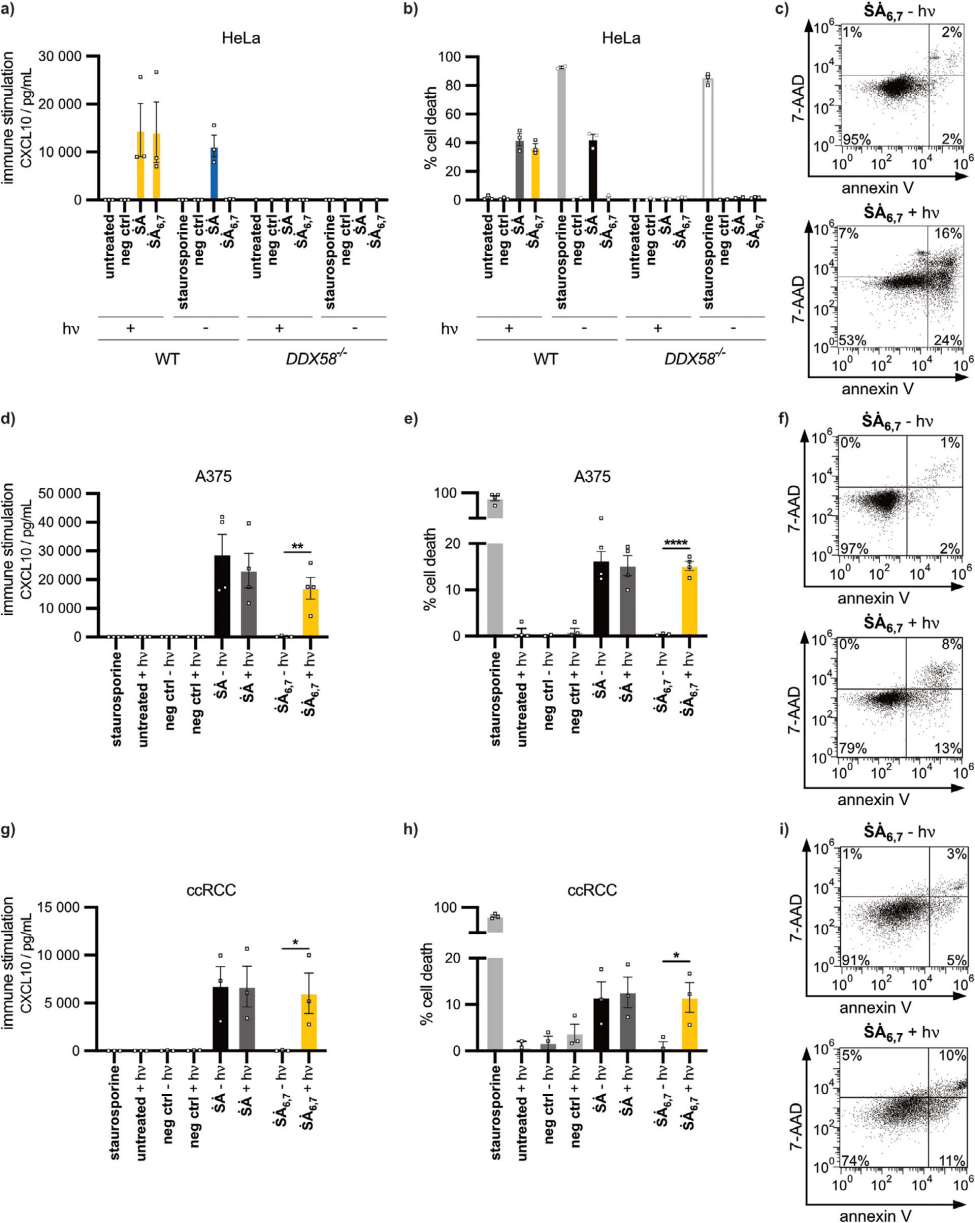
Photocaged RIG‐I ligand enables light‐mediated control of immune stimulation and cell death induction in tumor cells. a–c) HeLa wildtype (WT) or HeLa RIG‐I knockout (DDX58*^−/−^*) cells were transfected with photocaged **ṠȦ_6,7_**, **ṠȦ** positive control or negative control RNA (800 ng mL^−1^) for 72 h. Following transfection cells indicated “+ hν” were irradiated with LED (365 nm, 2 mW) for 10 min. a) CXCL10 induction was measured in the supernatant by ELISA. b) Percentage of cell death as determined by FACS (Fluorescence Activated Cell Sorting) analysis of annexin V/7‐AAD staining. c) FACS gating for **ṠȦ_6,7_** treated HeLa wildtype cells with and without irradiation. d–f) Human melanoma cells (A375) were treated as described for HeLa cells. g–i) Human primary renal cancer cells ccRCC were treated as described for HeLa cells. a), b), d), e), g), h) Results show the means ± SEM of at least three independent experiments. Two‐tailed unpaired *t*‐test, *P ≤ 0.05, **P ≤ 0.01, ****P ≤ 0.0001 c), f), i) Results are representative of at least three independent experiments. Conditions with no values indicated were below the detection limit of the assay.

Localized RIG‐I activation in tumor tissue holds promise for immunotherapy of spatially confined superficial cancer entities, such as skin melanoma. To demonstrate the utility of the newly developed photocaged RIG‐I ligand (**ṠȦ_6,7_**) for the induction of light‐controlled tumor cell death, we tested a number of tumor cell lines, and primary human tumor cells (Figure 4).

First, WT and RIG‐I (*DDX58^−/−^)*‐deficient HeLa cells, a human cervical carcinoma cell line, were used to demonstrate that the effects of the photocaged ligand were indeed RIG‐I dependent and in particular to exclude nonspecific effects of photocage chemistry on IFN induction and cell death in tumor cells. In HeLa WT cells, the positive control **ṠȦ** and the photocaged RNA **ṠȦ_6,7_** (both at 800 ng mL^−1^) combined with subsequent light irradiation induced a robust CXCL10 response (Figure 4a) and substantial tumor cell death as measured by annexin V and 7‐AAD uptake (Figure 4b,c) whereas irradiation of untreated or control transfected cells did not lead to cell death. In line with the specificity of our photocage ligand, HeLa RIG‐I knockout cells showed no CXCL10 response, and no increase in cell death was detectable after **ṠȦ_6,7_** irradiation (Figure 4b) although annexin V and 7‐AAD uptake could still be induced by staurosporine. Furthermore, cells that were transfected with **ṠȦ_6,7_** but not exposed to light showed no increase in cytokine production or cell death (Figure 4a–c), supporting the utility of **ṠȦ_6,7_** to control RIG‐I activation in tumor cells.

Light‐controlled activation of RIG‐I ligands is limited to tissues accessible by light at the energy levels required to uncage the ligand. Depending on its intensity, UV‐light (UV‐A) penetrates skin relatively well. UV‐A combined with psoralen (PUVA) has been in use for more than 50 years to treat certain dermal conditions such as psoriasis.^[^ ^42^, ^43^ ^]^ Lucas et al. successfully demonstrated the activation of photocaged antimiRs in mouse skin by UV‐light of similar wavelength (385 nm).^[^ ^44^ ^]^ Early‐stage melanoma is a directly accessible superficial type of cancer since the tumor is confined in the upper layer of the skin. As a proof of principle, human melanoma cells (A375) were transfected with **ṠȦ_6,7_**, **ṠȦ** or negative control RNA (800 ng mL^−1^). Both the positive control **ṠȦ** and the photocaged ligand **ṠȦ_6,7_** combined with UV‐light exposure (but not without) induced CXCL10 (Figure 4d) and cell death (Figure 4e). Irradiation alone had no effect on melanoma cells.

We then studied the response of primary human carcinoma cells derived from tumor tissue of a patient with clear cell renal cell carcinoma (ccRCC). Again, cells transfected with the photocaged ligand **ṠȦ_6,7_** (800 ng mL^−1^) demonstrated highly significant CXCL10 induction and cell death that was strictly dependent on exposure of cells to UV‐light (Figure 4g‐i). The lack of any UV‐independent response of cells, even after a 72 h incubation period, argues against spontaneous uncaging of **ṠȦ_6,7_** in the tumor environment, highlighting the chemical stability of the newly developed photocaged RIG‐I ligand.

## Conclusion

In this study, we engineered a photocaged ligand for RIG‐I that is activated by exposure to light. While NPE (1‐(2‐nitrophenyl) ethyl)‐derived caging groups have been known for decades and utilize well‐established techniques, with this work, we have paved the way to novel approaches for the photopharmacological spatio‐temporal control of innate immune signaling in a clinical context, with the aim of reducing unwanted systemic side effects through the application light‐controlled, precise therapeutic interventions. Important features of this novel photocaged ligand are a complete off‐state even at high concentrations (tested up to 1600 ng mL^−1^ or 100 nM) and the RIG‐I specificity of the activated ligand. The design of this ligand was achieved by a 2´‐*O*‐methylation screen of the antisense strand which demonstrated that the critical sequence comprises A_5_ to A_8_, with the strongest effect for A_6_ and A_7_. The attachment of two photolabile NPE groups to A_6_ and A_7_ eliminated the ability of the ligand to activate RIG‐I.

It is interesting to note that in published crystal structures of RIG‐I bound to dsRNA, the amino acid E510 in the Hel2i domain of RIG‐I interacts with the 2´‐hydroxy group of exactly that nucleoside position that corresponds to A_7_ in our dsRNA ligand SA.^[^ ^34^, ^35^ ^]^ E510 has been postulated to act as a gatekeeper of the Hel2i interaction with CARD2 in the non‐active autoinhibited form of RIG‐I (apo‐RIG‐I). A RIG‐I E510 V mutant was reported to induce an interferonopathy (Singleton Merten‐Syndrome) in patients. This was explained by aberrant proofreading activity of endogenous RNA with more frequent CARD release.^[^ ^44^ ^]^ 2′‐*O*‐Methyl modification of A_7_ could potentially inhibit E510 binding thereby preventing CARD2 domain release upon dsRNA binding and thus RIG‐I activation. Our results for the first time provide evidence that binding of RIG‐I to the 2′‐hydroxy backbone of this RNA region is in fact necessary for RIG‐I activation, and that in addition to the key position A_7_, the neighboring A_5_, A_6,_ and A_8_ contribute to RIG‐I binding as well, thus providing novel possible targets for RNA backbone modifications to control RIG‐I activity. The exact positions applicable for steric hindrance of binding may vary with the RNA sequence of the RIG‐I ligand, leaving room for future systematic analyses of sequence dependence.

The complete off‐state in our novel photocaged RIG‐I ligand is achieved by combining two photolabile groups at A_6_ and A_7_ of the antisense strand each of which already provides strong inhibition of RIG‐I activity. The fact that one photolabile group at the S_1_ position of the sense strand is not sufficient for a complete off‐state despite a complete blockade of binding if this position is modified by 2´‐*O*‐methyl strongly suggests that unlike the 2´‐*O*‐methyl, a small percentage of the photolabile group is cleaved off even under normal light conditions. This assumption is supported by our LC‐MS analysis which showed a small percentage of uncaged ligand, which might be sufficient to cause the small RIG‐I activating effect that counteracts the desired complete off‐state.

Exposure to light cleaves off the photolabile groups thereby turning on the RIG‐I ligand. This RIG‐I ligand mimics the presence of viral RNA and triggers potent antiviral pathways in vivo. Thus, the targeted application of light can now determine the time and location of RIG‐I activation, enabling a precise spatiotemporal control of innate antiviral responses. Besides the possibility of localized antiviral treatment, the photocaged ligand now can be used to address one of the key challenges of cancer immunotherapy: the targeted administration of a powerful innate immune stimulus to the tumor tissue. Although the photocaged ligand can up to now only be activated in superficial tumors in surfaces accessible to UV‐light, skin cancer including melanoma are obvious targets for such a therapeutic strategy. High doses of the photocaged ligands can be applied by established areal injection methods in vivo and then activated by light specifically at the circumscribed site of the tumor.^[^ ^45^ ^]^ Tumor‐targeted gamma‐irradiation and MAP kinase inhibition synergize with RIG‐I activation,^[^ ^23^, ^24^ ^]^ further enhancing the combinatorial arsenal for effective immunotherapy of skin cancer.

In the current literature, the combination of different modifications such as 2′‐fluoro, 2′‐methyl and phosphorothioate modifications have been used to improve nuclease stability and the immunological properties of potential therapeutic oligonucleotides. For example, the introduction of 2′‐sugar‐modifications has been used to prevent RNase‐mediated degradation of oligoribonucleotides into degradation products including the monomeric uridine which is required for TLR8 activation.^[^ ^46^, ^47^ ^]^ In contrast, in this work, we have identified novel specific nucleotide positions that are critical for precise accommodation of the RNA oligonucleotide ligand into the binding pocket of RIG‐I. These previously unknown interaction sites of the RIG‐I‐RNA binding now for the first time allowed the complete blockade of RIG‐I activation by the site‐specific placement of caging groups at these sites of the RNA. This exemplifies the potential of combined efforts of chemical and immunological approaches for developing oligonucleotide‐based precise control of innate immunity. Notably, the concentrations of the photocaged ligand used here (1.28–1600 ng mL^−1^, equivalent to 0.08–100 nM) are in the same range as reported for several siRNA (short interfering RNA) used for gene silencing (1.5–80 nM),^[^ ^48^ ^]^ including some that are now approved for clinical use.^[^ ^49^ ^]^

To our knowledge, this study presents the first photocaged nucleic acid ligand of an innate immune receptor. Innate immune receptors sense a broad range of microbial molecules, provide alarm signals and trigger early defense mechanisms against invading pathogens. Similar to the RIG‐I ligand in this work, other agonists of innate immune receptors can provide a molecular toolbox for targeted activation of a broad range of innate immune receptors. The controlled activation of these signals could be used to strengthen innate antimicrobial defense or to provide optimal activation of adaptive immunity, such as vaccine adjuvants. We are currently developing new protocols for photochemical control of oligonucleotides in the (red) phototherapeutic window which will further expand the applications of photocaged innate immune ligands. Therefore, photocaging has the potential to open a new chapter in the precise manipulation of innate immunity in both experimental settings and therapeutic applications.

## Supporting Information

The authors have cited additional references within the Supporting Information.^[^ ^39^, ^40^, ^50^ ^]^

## Conflict of Interests

The authors declare no conflict of interest.

## Supporting information



Supporting Information

## Data Availability

The data that support the findings of this study are available from the corresponding author upon reasonable request.
