# Supplementary material for: Immunoengineering of a Photocaged 5´‐triphosphate Oligoribonucleotide Ligand for Spatiotemporal Control of RIG‐I Activation in Cancer
Source: Angew Chem Int Ed Engl. 2025 Apr 21;64(21):e202423321. doi: 10.1002/anie.202423321 (PMC12087816; doi:10.1002/anie.202423321)
Supplement: Supplementary file 1 — Supporting Information [file ANIE-64-e202423321-s001.docx]

Supporting Information
©Wiley-VCH 2021
69451 Weinheim, Germany

Immunoengineering of a Photocaged 5´-triphosphate Oligoribonucleotide Ligand for Spatiotemporal Control of RIG-I Activation in Cancer

Sandra Anika Lewash^[a]+^, Vivien Rose McKenney^[b]+^, Christine Wuebben^[a]^, Janos Ludwig^[a]^, Racha Hosni^[d]^, Dirk Radzey^[a]^, Marieta I. Toma^[d]^, Eva Bartok^[c]^, Martin Schlee^[a]^, Thomas Zillinger^[a,e]^, Alexander Heckel^[b]^*, Gunther Hartmann^[a]^*

**Abstract:** Retinoic acid-inducible gene I (RIG-I) is a cytoplasmic innate immune receptor that triggers antiviral responses upon detection of viral RNA. RIG-I can be specifically activated by short double-stranded(ds) RNA with a blunt 5’ end bearing a triphosphate, mimicking nascent viral transcripts. RIG-I is expressed in all nucleated cells, including most tumor cells (e.g., melanoma). Tumor cells are specifically sensitive to RIG-I-induced cell death. RIG-I ligands have been developed for cancer immunotherapy, but tumor-specific targeting remains challenging. Here we developed a highly potent oligonucleotide ligand for spatiotemporally controlled activation of RIG-I by light exposure. Through structural considerations and functional studies we identified a combination of two specific positions in a RIG-I oligonucleotide ligand for which the substitution of both respective 2′-hydroxy groups of the ribose by photolabile protecting groups (2′-photocages) resulted in a complete loss of RIG-I ligand activity, whereas photocaging the individual positions was not sufficient to turn off RIG-I. Light exposure fully restored RIG-I activation by the photocaged RIG-I ligand, enabling light-controlled RIG-I-mediated cell death of human melanoma cells and human primary renal cell carcinoma which had internalized the photocaged RIG-I ligand prior to light exposure. This novel photoswitchable RIG-I oligonucleotide ligand may be applicable for precise light-controlled RIG-I activation in superficial tumors such as melanoma.

DOI: 10.1002/anie.2024XXXXX

Table of Contents

[1. Chemical Synthesis 2](#_Toc182229204)

[1.1 Materials and Methods 2](#_Toc182229205)

[1.2 Synthesis of 2’-photolabile protected phosphoramidites 2](#_Toc182229206)

[2. Oligonucleotide Synthesis 6](#_Toc182229208)

[2.1 Materials and Methods 6](#_Toc182229209)

[2.2 Purification 7](#_Toc182229210)

[2.3 Mass spectrometry 7](#_Toc182229211)

[2.4 Experimental irradiation of Ȧ_6,7_ 10](#_Toc182229212)

[3. Cell experiments to measure RIG-I activity and RIG-I induced apoptosis 12](#_Toc182229213)

[3.1 Materials and Methods 12](#_Toc182229214)

[3.2 Irradiation of photolabile RNA-ligands in cells 13](#_Toc182229215)

[3.3 Titration of irradiation time 14](#_Toc182229216)

[4. NMR-Spectra 15](#_Toc182229217)

[5. ESI-HRMS mass-Spectra 23](#_Toc182229218)

[6. References 25](#_Toc182229219)

[7. Author Contributions 25](#_Toc182229220)

1. Chemical Synthesis

1.1 Materials and Methods

All reactions were performed under argon atmosphere using dry solvents purchased from *Acros Organics*. Deionised (DI) water was used for all experiments. Reagents were purchased from *Sigma Aldrich*, *TCI*, *BLDPharm* and *abcr* and used without further purification.

Reaction progress was monitored using silica-gel 60-coated TLC-sheets. For purification by column chromatography silica gel 60 (0.04 – 0.06 mm) was used. For purification via flash column chromatography a puriFlash® XS 420 by *Interchim* was used.

^1^H-, ^13^C- and ^31^P-NMR-spectra were recorded in dimethyl sulfoxide-d6 (DMSO-*d6*) (reference ^1^H = 2.50 ppm, ^13^C = 39.52 ppm) with Bruker Avance AV500 MHz spectrometer. High-resolution mass spectroscopy was obtained on a *Bruker* MicroTof-qII (ESI-HRMS).

1.2 Synthesis of 2’-photolabile protected phosphoramidites

The coupling of the photolabile protecting group to the 2′-O of the ribonucleoside followed the literature known synthesis route of Pitsch et al.^[1]^

***N^6^*-Benzoyl-5’-*O*-[bis(4-methoxyphenyl)phenylmethyl]-2′**-**O**-**[[(R,S)**-**1-(2-nitrophenyl)ethoxy]methyl]adenosine (2a)**

**Compound 1a (1.00 g, 1.48 mmol, 1.00 eq.) was dissolved in 4 mL dry 1,2-dichloroethane, *N,N*-diisopropylethylamine (0.91 mL, 5.19 mmol, 3.50 eq) and dibutyltinn-dichloride (0.51 g, 1.66 mmol, 1.10 eq.) were added to the solution and stirred for 1 h at room temperature. The reaction mixture was heated to 80 °C and compound *R,S*-1-(1-(chloromethoxy)ethyl)-2-nitrobenzene (0.42 g, 1.93 mmol, 1.30 eq) in 1 mL dry 1,2-dichlorethane was added. The solution was stirred for 30 min at 80 °C. Sat. NaHCO_3_-solution was added and the mixture was stirred for 30 min at room temperature. The aqueous phase was extracted three times with 20 mL CH_2_Cl_2_ and the organic layer was dried over Na_2_SO_4_. The solvent was removed under reduced pressure. The crude product was purified by flash column chromatography (**CH_2_Cl_2_**/MeOH = 100:0 to 98:2 v/v, the silica gel was deactivated with 2% Et_3_N). Compound 2a, a yellow foam, was obtained as a diastereomeric mixture.**

**Yield: 0.42 g (0.49 mmol), 33%.**

**TLC: R_f_ = 0.56 (CH_2_Cl_2_/EtOAc = 5:1 v/v).**

**^1^H-NMR: (500 MHz; DMSO-*d_6_*, 298 K, TMS) δ (ppm) =** 11.24 (s, 1H, N*H*), 11.20 (s, 1H, N*H*), 8.69 (s, 1H, *H*-8), 8.64 (s, 1H, *H*-2), 8.63 (s, 1H, *H*-2), 8.48 (s, 1H, *H*-8), 8.08 - 8.05 (m, 4H, 2x H_Ar_ Benzyl), 7.89 (dd, *J* = 8.2, 1.2 Hz, 1H, H_Ar_ NPE), 7.79 (dd, *J* = 8.2, 1.2 Hz, 1H, H_Ar_ NPE), 7.68 - 7.62 (m, 3H, H_Ar_ NPE + 2x H_Ar_ Benzyl), 7.59 - 7.53 (m, 6H, 2x H_Ar_ 2x Benzyl + 2x H_Ar_ NPE), 7.50 – 7.47 (m, 1H, H_Ar_ NPE), 7.42 – 7.37 (m, 3H, H_Ar_ NPE + DMTr-H), 7.34 – 7.18 (m, 18H, H_Ar_ NPE + DMTr-H, 6.86 – 6.80 (m, 8H, 2x DMTr-H), 6.22 (d, *J* = 5.3 Hz, 1H, 1’-*H*), 5.93 (d, *J* = 4.2 Hz, 1H, 1’-*H*), 5.49 (d, *J* = 6.2 Hz, 1H, 3’-O*H*), 5.38 (d, *J* = 5.7 Hz, 1H, 3’-O*H*), 5.25 (q, *J* = 6.4 Hz, 1H, -OC*H*CH_3_), 5.07 – 5.01 (m, 2H, 2’-*H* + *-*OC*H*CH_3_), 4.97 (t, *J* = 4.6 Hz, 1H, 2’-*H*), 4.86 (d, *J* = 7.1 Hz, 1H, -OC*H*_2_O), 4.80 (d, *J* = 7.2 Hz, 1H, -OC*H*_2_O), 4.76 (d, *J* = 7.2 Hz, 1H, -OC*H*_2_O), 4.58 – 4.53 (m, 1H, 3’-*H* & -OC*H*_2_O), 4.44 (q, *J* = 5.2 Hz, 1H, 3’-*H*), 4.15 (q, *J* = 4.4 Hz, 1H, 4’-*H*), 4.10 – 4.07 (m, 1H, 4’-*H*), 3.72 – 3.71(m, 12H, 2x -OC*H*_3_), 3.29 – 3.18 (m, 4H, 2x 5’-*H*), 1.40 (d, *J* = 6.4 Hz, 3H, -CHC*H*_3_), 1.15 (d, *J* = 6.4 Hz, 3H, -CHC*H*_3_).

**^13^C{1H}-NMR:** (120 MHz; DMSO**-*d_6_***, 298 K, TMS) **δ [ppm] = 166.1, 166.1, 158.6, 158.5, 152.5, 152.2, 152.1, 152.0, 151.0, 150.9, 148.3, 147.6, 145.3, 143.9, 143.8, 138.8, 138.3, 136.0, 136.0, 135.9, 135.9, 134.1, 133.9, 133.9, 133.7, 132.9, 130.2, 130.1, 129.1, 129.0, 128.9, 128.9, 128.7, 128.3, 128.2, 128.1, 128.1, 128.0, 127.2, 127.1, 126.4, 126.3, 124.4, 124.2, 113.6, 113.6, 93.5, 93.0, 87.1, 87.0, 86.1, 86.0, 84.4, 83.8, 78.0, 77.6, 70.6, 70.1, 70.0, 69.6, 64.0, 63.7, 55.5, 55.4, 23.7, 23.4.**

**ESI-HRMS: *m/z* calculated for C_47_H_44_N_6_O_10_ [M+H]^+^ 853.3192, found 853.3198 (Δ_m_ = 0.0006, Δ_m_/m = 0.738 ppm).**

***N^6^*-Benzoyl-5’-*O*-[bis(4-methoxyphenyl)phenylmethyl]-2′**-**O**-**[[(R,S)-1-(2-nitrophenyl)ethoxy]methyl]adenosine-(2-cyanoethyl)diisopropylphosphoramidite (3a)**

**Compound 2a (0.17 g, 0.20 mmol, 1.00 eq.) and *N,N*-diisopropylethylamine (0.89 mL, 0.40 mmol, 5.00 eq.) were dissolved in a microwave vessel in 3.5 mL dry MeCN and the solution was degassed for 15 min. *N,N*-2-cyanoethyl-*N,N*‘-diisopropylchlorophosphoramidite (0.17 mL, 1.00 mmol, 2.00 eq.) was added and the reaction mixture was microwaved for 1.5 h at 40 °C. The reaction was terminated by adding 5 mL 5% NaHCO_3_-solution. The aqueous phase was extracted three times with 5 mL EtOAc. The organic layer was dried over Na_2_SO_4_ and the solvent was removed under reduced pressure. The crude product was purified via column chromatography (Cyclohexane/EtOAc = 1:2 to 1:4 v/v, the silica gel was deactivated with 2% Et_3_N). Compound 3a, a yellow foam, was obtained as a diastereomeric mixture.**

**Yield: 0.09 g (85.46 μmol), 45%.**

**TLC: R_f_ = 0.48 (Cyclohexane/EtOAc = 1:4 v/v).**

**^1^H-NMR: (500 MHz; DMSO-*d_6_*, 298 K, TMS) δ (ppm) =** 11.23 (s, 1H, N*H*), 11.18 (s, 1H, 2x N*H*), 8.67 (s, 1H, *H*-8), 8.62 (s, 1H, *H*-2), 8.53 (s, 1H, *H*-2), 8.46 (s, 1H, *H*-8), 8.05 - 8.04 (m, 4H, 4x H_Ar_ Benzyl), 7.90 (dd, *J* = 1.0 Hz, 8.1 Hz, 1H, H_Ar_ NPE), 7.82 (dd, *J* = 1.1 Hz, 8.1 Hz, 1H, H_Ar_ NPE), 7.67 - 7.62 (m, 3H, 2x H_Ar_ Benzyl & H_Ar_ NPE), 7.58 – 7.52 (m, 5H, 4x H_Ar_ Benzyl & H_Ar_ NPE), 7.50 - 7.47 (m, 1H, H_Ar_ NPE), 7.45 - 7.30 (m, 7H, 3x H_Ar_ NPE & 4x H_Ar_ DMTr), 7.26 - 7.16 (m, 14H, H_Ar_ DMTr), 6.85 - 6.78 (m, 8H, H_Ar_ DMTr), 6.21 (d, *J* = 5.6 Hz, 1H, 1’-*H*), 5.96 (d, *J* = 4.8 Hz, 1H, 1’-*H*), 5.30 (t, *J* = 5.4 Hz, 1H, 2’-*H*), 5.24 (t, *J* = 4.9 Hz, 1H, 2’-*H*), 5.19 (q, *J* = 6.4 Hz, 1H, -C*H*CH_3_), 4.99 (q, *J* = 6.1 Hz, 1H, -C*H*CH_3_), 4.81 – 4.72 (m, 4H, 3’-*H* & 1.5x -OC*H*_2_O-), 4.67 – 4.63 (m, 1H, 3’-*H*), 4.52 (d, *J* = 7.2 Hz, 1H, -OC*H*_2_O-), 4.22 (q, *J* = 4.6 Hz, 1H, 4’-*H*), 4.18 (q, *J* = 4.6 Hz, 1H, 4’-*H*), 3.90 – 3.71 (m, 16H, 2x -OC*H*_2_CH_2_CN & 4x –OC*H*_3_), 3.60 - 3.49 (m, 4H, 2x -PN(C*H*(CH_3_)_2_)_2_), 3.38 - 3.24 (m, 45H, 3x 5’-*H* & H_2_O), 3.18 - 3.14 (m, 1H, 5’-*H*), 2.81 - 2.79 (m, 2H, -OCH_2_C*H*_2_CN), 2.81 - 2.79 (m, 2H, -OCH_2_C*H*_2_CN), 1.36 (d, *J* = 6.3 Hz, 3H, -CHC*H*_3_), 1.21 (d, *J* = 6.5 Hz, 3H, -CHC*H*_3_), 1.16 - 0.99 (m, 27H, 2x -PN(CH(C*H*_3_)_2_)_2_).

**^13^C{1H}-NMR:** (120 MHz; DMSO**-*d_6_***, 298 K) **δ [ppm] = 165.6, 158.1, 158.1, 158.1, 158.0, 151.8, 151.8, 151.6, 151.4, 150.6, 150.5, 147.7, 147.0, 144.7, 144.0, 143.8, 138.2, 137.8, 135.4, 135.3, 135.3, 133.7, 133.4, 132.5, 129.7, 129.6, 128.6, 128.5, 128.5, 128.4, 127.8, 127.7, 127.7, 127.6, 127.6, 127.4, 126.7, 126.6, 126.1, 125.9, 124.0, 123.8, 118.9, 115.6, 113.1, 113.1, 113.1, 113.0, 92.8, 86.9, 86.9, 85.7, 85.6, 82.5, 82.4, 76.5, 76.0, 71.2, 71.0, 70.7, 70.6, 70.0, 69.9, 69.7, 63.0, 62.7, 58.9, 58.9, 58.8, 58.7, 55.0, 46.3, 45.7, 45.6, 42.7, 42.6, 42.6, 42.5, 29.1, 29.1, 29.0, 29.0, 28.9, 28.8, 28.7, 28.7, 28.7, 28.6, 28.6, 28.5, 28.5, 26.6, 26.5, 25.1, 24.5, 24.3, 24.3, 24.2, 24.2, 24.1, 23.1, 23.0, 22.9, 22.2, 22.1, 22.0, 19.9, 19.9, 19.8, 19.8, 18.8.**

**^31^P{^1^H}-NMR: (202 MHz; DMSO-*d_6_*, 298 K) δ [ppm] = 149.6, 149.5.**

**ESI-HRMS: *m/z* calculated for C_56_H_61_N_8_O_11_P [M+H]^+^ 1053.4270, found 1053.4292 (Δ_m_ = 0.0022, Δ_m_/m = 2.069 ppm).**

**5′-O-[bis(4-methoxyphenyl)phenylmethyl]-N^2^-[(dimethylamino)methylene]-2′-O-[[(R,S)-1-(2-nitrophenyl)ethoxy]methyl]guanosine (2b)**

**Compound 1b (0.50 g, 0.78 mmol, 1.00 eq.) was dissolved in 4 mL dry. 1,2-dichloroethane, *N,N*-diisopropylethylamine (0.66 mL, 3.90 mmol, 5.00 eq) and dibutyltinn-dichloride (0.28 g, 0.94 mmol, 1.20 eq.) were added to the solution and stirred for 1 h at room temperature. The reaction mixture was heated to 80 °C and compound *R,S*-1-(1-(chloromethoxy)ethyl)-2-nitrobenzene (0.18 g, 0.82 mmol, 1.05 eq) in 1 mL 1,2-dichloroethane was added. The solution was stirred for 30 min at 80 °C. Sat. NaHCO_3_-solution was added and the mixture was stirred for 30 min at room temperature. The aqueous phase was extracted three times with 15 mL CH_2_Cl_2_ and the organic layer was dried over Na_2_SO_4_. The solvent was removed under reduced pressure. The crude product was purified by flash column chromatography (CH_2_Cl_2_/MeOH = 100:0 to 97:3 v/v, the silica gel was deactivated with 2% Et_3_N). Compound 2b, a yellow foam, was obtained as a diastereomeric mixture.**

**Yield: 0.11 g (1.30 mmol), 17%.**

**TLC: R_f_ = 0.68 (CH_2_Cl_2_/MeOH = 50:1 v/v).**

**^1^H-NMR: (500 MHz; DMSO-*d_6_*, 298 K, TMS) δ (ppm) = 11.37 (s, 1H, N*H*), 11.34 (s, 1H, N*H*), 8.51 (s, 1H, -NC*H*N(CH_3_)_2_), 8.46 (s, 1H, -NC*H*N(CH_3_)_2_), 7.94 (s, 1H, *H*-8), 7.90 (dd, *J* = 8.1, 1.1 Hz, 1H, H_Ar_ NPE), 7.83 (dd, J = 8.2, 1.1 Hz, 1H, H_Ar_ NPE), 7.80 (s, 1H, *H*-8), 7.70 – 7.66 (td, J = 7.9, 1.2 Hz, 1H, H_Ar_ NPE), 7.62 – 7.59 (m, 2H, H_Ar_ NPE), 7.52 – 7.49 (m, 2H, H_Ar_ NPE), 7.38 – 7.32 (m, 5H, H_Ar_ NPE + 2x DMTr*-H*), 7.29 – 7.19 (m, 14H, 2x DMTr-*H*), 6.86 – 6.80 (m, 8H, 2x DMTr-*H*), 6.01 (d, *J* = 5.3 Hz, 1H, 1’-*H*), 5.74 (d, *J* = 4.1 Hz, 1H, 1’-*H*), 5.39 (d, *J* = 6.3 Hz, 1H, 3’-O*H*), 5.27 – 5.24 (m, 2H, 3’-O*H* & -OC*H*CH_3_), 5.06 (q, *J* = 6.4 Hz, 1H, -OC*H*CH_3_), 4.84 (d, *J* = 7.1 Hz, 1H, -OC*H*_2_O), 4.77 (d, *J* = 7.4 Hz, 1H, -OC*H*_2_O), 4.70 – 4.65 (m, 3H, -OC*H*_2_O & 2’-*H*), 4.49 (d, J = 7.3 Hz, 1H, -OC*H*_2_O), 4.41 (q, *J* = 5.6 Hz, 1H, 3’-*H*), 4.30 (q, *J* = 5.1 Hz, 1H, 3’-*H*), 4.02 – 3.97 (m, 2H, 2x 4’-*H*), 3.73 (s, 6H, -OC*H*_3_), 3.72 (s, 6H, -OC*H*_3_), 3.26 – 3.16 (m, 4H, 2x 5’-*H*), 3.09 (d, *J* = 1.9 Hz, 6H, -CHN(C*H*_3_)_2_), 3.04 (d, *J* = 1.9 Hz, 6H, -CHN(C*H*_3_)_2_) 1.41 (d, *J* = 6.6 Hz, 3H, -CHC*H*_3_), 1.2 (d, *J* = 6.2 Hz, 3H, -CHC*H*_3_).**

**^13^C{1H}-NMR:** (120 MHz; DMSO**-*d_6_***, 298 K) **δ [ppm] = 158.1, 158.1, 158.0, 158.0, 157.9, 157.8, 157.6, 157.5, 157.4, 157.2, 149.9, 149.6, 148.0, 147.3, 144.8, 144.7, 138.3, 137.7, 136.3, 136.2, 135.5, 135.4, 135.4, 133.7, 133.4, 129.7, 129.7, 129.6, 128.7, 128.3, 127.8, 127.8, 127.7, 127.6, 127.6, 126.7, 126.6, 124.0, 123.9, 119.8, 119.7, 113.2, 113.1, 92.9, 92.3, 85.6, 85.4, 85.4, 85.2, 83.5, 82.9, 78.6, 78.0, 70.0, 69.6, 69.2, 69.0, 63.8, 63.5, 55.0, 55.0, 34.7, 23.3, 22.9.**

**ESI-HRMS: *m/z* calculated for C_43_H_45_N_7_O_10_ [M+H]^+^, 820.3301, found 820.3330 (Δ_m_ = 0.0029, Δ_m_/m = 3.572 ppm).**

**5′-O-[bis(4-methoxyphenyl)phenylmethyl]-N^2^-[(dimethylamino)methylene]-2′-O-[[(R,S)-1-(2-nitrophenyl)ethoxy]methyl]guanosine-(2-cyanoethyl)diisopropylphosphoramidite (3b)**

**Compound 2b (0.10 g, 0.12 mmol, 1.00 eq.) and *N,N*-diisopropylethylamine (0.10 mL, 0.61 mmol, 5.00 eq.) were dissolved in a microwave vessel in 3.5 mL dry CH_2_Cl_2_ and the solution was degassed for 15 min. *N,N*-2-cyanoethyl-*N,N*‘-diisopropylchlorophosphoramidite (0.05 mL, 0.24 mmol, 2.00 eq.) was added and the reaction mixture was microwaved for 1.5 h at 40 °C. 5 mL 5% NaHCO_3_-solution were added to the solution and the aqueous phase was extracted three times with 5 mL EtOAc. The organic layer was dried over Na_2_SO_4_ and the solvent was removed under reduced pressure. The crude product was purified via column chromatography (CH_2_Cl_2_/acetone = 100:0 to 6:4 v/v, the silica gel was deactivated with 2% Et_3_N). Compound 3b, a yellow foam, was obtained as a diastereomeric mixture.**

**Yield: 0.08 g (0.08 mmol), 67%.**

**TLC: R_f_ = 0.41 (CH_2_Cl_2_/acetone = 6:4 v/v).**

**^1^H-NMR: (500 MHz; DMSO-*d_6_*, 298 K, TMS) δ (ppm) = 11.40 - 11.39 (m, 1H, N*H*), 8.46 (s, 1H, NC*H*NCH_3_), 7.89 - 7.87 (m, 1H, *H*-8), 7.92 - 7.86 (m, 1H, H_Ar_ NPE), 7.68 - 7.64 (m, 1H, H_Ar_ NPE), 7.60 - 7.55 (m, 1H, H_Ar_ NPE), 7.53 - 7.49 (m, 1H, H_Ar_ NPE), 7.38 - 7.34 (m, 2H, H_Ar_ DMTr), 7.29 - 7.21 (m, 7H, H_Ar_ DMTr), 6.86 - 6.80 (m, 4H, H_Ar_ DMTr), 6.03 - 6.00 (m, 1H, 1’-*H*), 5.04 - 4.88 (m, 2H, -OC*H*CH_3_ & 2’-*H*), 4.78 - 4.70 (m, 1H, -OC*H*_2_O), 4.55 - 4.43 (m, 2H, -OC*H*_2_O & 3’-*H*), 4.16 - 4.09 (m, 1H, 4’-*H*), 3.75 - 3.64 (m, 7H, -OC*H*_3_ & -POC*H*_2_CH_2_CN), 3.59 - 3.44 (m, 3H, -POC*H*_2_CH_2_CN & -P(NC*H*(CH_3_)_3_)_2_), 3.27 - 3.22 (m, 2H, 5’-*H*), 3.07 - 3.06 (m, 3H, -NCHN(C*H*_3_)_2_), 3.03 - 3.01 (m 3H, -NCHN(C*H*_3_)_2_), 2.73 - 2.52 (m, 6H, -POCH_2_C*H*_2_CN & DMSO), 1.16 - 0.91 (m ,12H, -CHC*H*_3_ & -P(NCH(C*H*_3_)_3_)_2_).**

**^13^C{^1^H}**-**NMR:** (120 MHz; DMSO**-*d_6_***, 298 K) **δ [ppm] = 158.1, 158.1, 157.9, 157.9, 157.6, 157.4, 157.4, 149.9, 148.0, 147.9, 144.7, 137.8, 136.7, 137.6, 136.7, 135.4, 135.3, 135.3, 133.9, 133.7, 133.7, 129.7, 129.7, 129.6, 129.6, 128.7, 128.7, 127.9, 127.8, 127.8, 127.7, 127.7, 127.6, 126.7, 126.7, 124.0, 124.0, 120.0, 119.9, 118.9, 113.2, 85.8, 85.7, 85.7, 69.6, 69.6, 58.7, 58.6, 55.0, 55.0, 42.6, 42.5, 41.6, 40.8, 40.6, 40.6, 34.6, 29.0, 28.9, 28.6, 26.0, 24.3, 24.2, 24.2, 24.1, 24.1, 23.0, 22.9, 22.1, 19.7, 19.7, 13.9,**

**^31^P{^1^H}-NMR: (202 MHz; DMSO-*d_6_*, 298 K) δ [ppm] = 149.6, 148.9.**

**ESI-HRMS: *m/z* calculated for C_52_H_62_N_9_O_11_P [M+H]^+^ 1020.4379, found 1020.4399 (Δ_m_ = 0.0020, Δ_m_/m = 1.940 ppm).**

**N^4^-Acetyl-5′-O-[bis(4-methoxyphenyl)phenylmethyl]-2′-O-[[(R,S)-1-(2-nitrophenyl)ethoxy]methyl]cytidine (2c)**

**Compound 1c (1.00 g, 1.70 mmol, 1.00 eq.) was dissolved in 4 mL dry 1,2-dichloroethane, *N,N*-diisopropylethylamine (1.04 mL, 5.96 mmol, 3.50 eq) and dibutyltinn-dichloride (0.58 g, 1.91 mmol, 1.12 eq.) were added to the solution and stirred for 1 h at room temperature. The reaction mixture was heated to 80 °C and compound *R,S*-1-(1-(chloromethoxy)ethyl)-2-nitrobenzene (0.48 g, 2.21 mmol, 1.30 eq) was added. The solution was stirred for 30 min at 80 °C. Sat. NaHCO_3_-solution was added and the mixture was stirred for 30 min at room temperature. The aqueous phase was extracted three times with 20 mL** CH_2_Cl_2_ **and the organic layer was dried over Na_2_SO_4_. The solvent was removed under reduced pressure and the crude product was purified via flash column chromatography (CH_2_Cl_2_/MeOH = 100:0 to 97:3 v/v, the silica gel was deactivated with 2% Et_3_N). Compund 2c, a yellow foam, was obtained as a diastereomeric mixture.**

**Yield: 0.47 g (0.48 mmol), 61%.**

**TLC: R_f_ = 0.42 (**CH_2_Cl_2_**/MeOH = 97:3 v/v).**

**^1^H-NMR: (500 MHz; DMSO-*d_6_*, 298 K) δ [ppm] = 10.91 (s, 2H, 2x N*H*), 8.27 (m, 2H, 2x *H*-6), 7.93 (m, 2H, 2x H_Ar_ NPE), 7.76 - 7.70 (m, 4H, 2x H_Ar_ NPE), 7.56 - 7.50 (m, 2H, 2x H_Ar_ NPE), 7.41 - 7.25 (m, 18H, H_Ar_ DMTr), 7.03 (dd, *J* = 7.0 Hz, 1.4 Hz, 1H, *H*-5), 7.00 (dd, *J* = 7.0 Hz, 1.4 Hz, 1H, *H*-5), 6.92 - 6.89 (m, 8H, H_Ar_ DMTr), 5.88 (bs, 1H, 1’-*H*), 5.74 (bs, 1H, 1’-H), 5.40 (q, *J* = 6.4 Hz, 1H, -OC*H*CH_3_), 5.34 - 5.30 (m, 2H, -OC*H*CH_3_ + 3’-O*H*), 5.20 (dd, J = 7.0 Hz, 1.4 Hz, 1H, 3’-O*H*), 5.06 (d, *J* = 6.9 Hz, 1H, -OC*H*_2_O), 4.90 (d, *J*= 6.9 Hz, 1H, -OC*H*_2_O), 4.68 (d, *J* = 6.9 Hz, 1H, -OC*H*_2_O), 4.59 (d, *J* = 6.9 Hz, 1H, -OC*H*_2_O), 4.36 - 4.32 (m, 1H, 3’-*H*), 4.25 - 4.21 (m, 1H, 3’-*H* ), 4.17 - 4.16 (m, 2H, 2x 2’-*H*), 4.03 - 3.99 (m, 2H, 2x 4’-*H*), 3.75 (s, 6H, -OC*H*_3_), 3.75 (s, 6H, -OC*H*_3_), 3.37 - 3.28 (m, 5H, 2x 5’-*H* + H_2_O), 2.11 (d, *J* = 1.9 Hz, 6H, 2x -NCOC*H*_3_), 1.47 - 1.44 (m, 6H, 2x -OCHC*H*_3_).**

**^13^C{^1^H}**-**NMR:** (120 MHz; DMSO**-*d_6_***, 298 K) **δ [ppm] = 171.0, 170.9, 162.4, 162.4, 158.2, 154.3, 154.2, 147.9, 147.9, 144.6, 144.5, 144.3, 138.4, 138.2, 135.4, 135.4, 135.1, 133.7, 129.8, 129.7, 128.6, 128.5, 128.0, 127.9, 127.9, 127.7, 126.8, 124.0, 124.0, 113.3, 95.4, 95.3, 92.0, 91.4, 89.3, 89.0, 86.0, 86.0, 82.1, 81.8, 78.5, 78.5, 69.4, 69.3, 67.9, 67.4, 61.8, 61.4, 55.0, 24.4, 24.3, 23.4, 23.0.**

**ESI-HRMS: *m/z* calculated for C_41_H_42_N_4_O_11_ [M+H]^+^ 767.2923, found 767.2959 (Δ_m_ = 0.0036, Δ_m_/m = 4.7 ppm).**

**N^4^-Acetyl-5′-O-[bis(4-methoxyphenyl)phenylmethyl]-2′-O-[[(R,S)-1-(2-nitrophenyl)ethoxy]methyl]cytidine-(2-cyanoethyl)diisopropylphosphoramidite (3c)**

**Compound 2c (0.17 g, 0.22 mmol, 1.00 eq.) and *N,N*-diisopropylethylamine (0.19 mL, 1.11 mmol, 5.00 eq.) were dissolved in a microwave vessel in 2 mL dry** MeCN **and the solution was degassed for 15 min. *N,N*-2-cyanoethyl-*N,N*‘-diisopropylchlorophosphoramidite (0.10 mL, 0.44 mmol, 2.00 eq.) was added and the reaction mixture was microwaved for 1 h at 40 °C. The reaction was terminated by adding 5 mL 5% NaHCO_3_-solution. The aqueous phase was extracted three times with5 mL** EtOAc**. The organic layer was dried over Na_2_SO_4_ and the solvent was removed under reduced pressure. The crude product was purified via column chromatography (**CH_2_Cl_2_**/acetone = 100:0 to 7:3 v/v, the silica gel was deactivated with 2% Et_3_N). Compund 3c, a yellow foam, was obtained as a diastereomeric mixture.**

**Yield: 0.09 g (93.1 μmol), 45%.**

**TLC: R_f_ = 0.73 (CH_2_Cl_2_/acetone = 7:3).**

**^1^H-NMR: (500 MHz; DMSO-*d_6_*, 298 K) δ [ppm] (Diastereomeres 1:0.6) = 10.93 (s, 1H, N*H*), 10.89 (s, 1H, N*H*), 8.33 (d, *J*** = 7.5 Hz, **1H, *H*-6), 8.27 (d, *J* = 7.5 Hz, 1H, *H*-6), 7.94 - 7.90 (m, 2H, 2x H_Ar_ NPE), 7.76 – 7.68 (m, 4H, 4x H_Ar_ NPE), 7.56 - 7.48 (m, 2H, 2x H_Ar_ NPE), 7.42 - 7.38 (m, 4H, 4x DMTr-*H*), 7.34 - 7.24 (m, 14H, 14x DMTr-*H*), 7.04 (d,** *J* = 7.5 Hz, **1H, *H*-5), 6.93 - 6.89 (m, 9H, 8x DMTr-H + *H*-5 ), 5.96 (d,** *J* = 2.3 Hz, **1H, 1’-*H*), 5.83 (d,** *J* = 2.1 Hz, **1H, 1’-*H*), 5.31 - 5.23 (m, 2H, 2x -OC*H*CH_3_), 5.06 (d,** *J* = 7.0 Hz, **1H, -OC*H*_2_O), 4.86 (d,** *J* = 6.9 Hz, **1H, -OC*H*_2_O ), 4.63 - 4.61 (m, 2H, 2x -OC*H*_2_O), 4.48 - 4.44 (m, 1H, 3’-*H*), 4.42 - 4.35 (m, 3H, 3’-*H* & 2x 2’-*H*), 4.16 - 4.12 (m, 2H, 4’-*H*), 3.75 - 3.67 (m, 14H, 4x -OC*H*_3_ & -OC*H*_2_CH_2_CN), 3.64 - 3.33 (m, 19H, -OC*H*_2_CH_2_CN & 2x -(NC*H*(CH_3_)_2_)_2_ & 4x 5’-*H* & H_2_O), 2.65 - 2.56 (m, 4H, 2x -OCH_2_C*H*_2_CN), 2.11 - 2.10 (m, 6H, 2x -NCOC*H*_3_), 1.47 (d,** *J* = 6.3 Hz, **3H, -CHC*H*_3_ ), 1.42 (d,** *J* = 6.3 Hz, **1H, -CHC*H*_3_), 1.25 - 0.96 (m, 24H, 2x -N(CH(C*H*_3_)_2_)_2_).**

**^13^C{^1^H}**-**NMR**: (120 MHz; DMSO**-*d_6_***, 298 K) **δ [ppm] = 171.9, 171.0, 170.9, 162.5, 162.4, 158.2, 154.3, 154.2, 147.9, 147.7, 144.5, 144.4, 144.3, 144.1, 138.3, 138.0, 135.1, 135.0, 135.0, 133.7, 133.6, 129.8, 128.7, 128.6, 128.0, 127.9, 127.8, 126.9, 124.0, 124.0, 118.7, 118.6, 113.2, 95.6, 95.5, 92.3, 91.7, 88.9, 88.6, 86.3, 86.2, 81.9, 81.5, 77.4, 70.0, 70.0, 69.5, 69.4, 68.9, 68.9, 61.3, 61.0, 58.2, 58.0, 55.0, 52.0, 46.2, 44.5, 44.5, 42.6, 42.5, 36.7, 34.7, 32.3, 31.3, 31.2, 28.9, 28.8, 28.8, 28.7, 28.6, 24.7, 24.4, 24.3, 24.3, 24.3, 24.2, 23.2, 23.1, 22.6, 22.1, 22.1, 19.9, 19.8, 19.8, 19.7, 19.4, 19.3, 18.8, 13.9, 7.2.**

**^31^P{^1^H}-NMR: (202 MHz; DMSO-*d_6_*, 298 K) δ [ppm] = 149.6, 149.5, 148.5, 148.0.**

**ESI-HRMS: *m/z* calculated for C_50_H_59_N_6_O_12_P [M+H]^+^ 967.4001, found 967.3995 (Δ_m_ = 0.0006, Δ_m_/m = 0.651 ppm).**

****2. Oligonucleotide Synthesis****

****2.1 Materials and Methods****

**The solid phase oligonucleotide synthesis was performed on an *ABI329 DNA/RNA synthesizer* from *Applied Bioscience* in 1 μmol scales. Commercially available phosphoramidites, CPG materials and reagents were used, purchased from *LGC Link*, *empBiotech, Sigma Aldrich* and *Glen Research*.**

**As activator 0.3 M BTT in acetonitrile was used with *UltraMild* capping reagents (tetrahydrofuran/pyridine/phenoxyacetic anhydride). 5’-Detritylation was performed using 3% TCA in CH_2_Cl_2_. For oxidation, 0.02 M iodine in pyridine/tetrahydrofuran/water (7:2:1) was used. Xanthene anhydride 0.2 M in pyridine/acetonitrile (9:1) was used as sulfurizing reagent. The coupling time for all RNA phosphoramidites was 12 min, for 2’-fluoro and 2’-methoxy phosphoramidites 6 min. The 2’-photolabile protected phosphoramidites 3a-c were coupled for 12 min. Ȧ_6_, Ȧ_7_ and Ȧ_6,7_ were synthesized DMTr-OFF, Ṡ_1_ was triphosphorylated in the final steps, which is described later on.**

**For all works involving oligonucleotides, RNAse free water was used. Therefore, 0.1% diethylpyrocarbonate (DEPC) was added to Milli-Q water, stirred overnight and autoclaved afterwards. Alternatively,** Ampuwa water was used.

**For Ȧ_6_, Ȧ_7_ and Ȧ_6,7_, solid supports containing a rC^Ac^ with 1000** Å pore size were used. To obtain a 5’-phosphorylated oligonucleotide, a chemical phosphorylation reagent (2-[2-(4,4'-Dimethoxytrityloxy)ethylsulfonyl]ethyl-(2-cyanoethyl)-(*N,N*-diisopropyl)-phosphoramidite) was coupled in the last step for 12 min. For **Ṡ_1_**, a solid support containing rU with 1000 Å pore size was used.

**Before cleavage of the solid-support, Ṡ1 had an additional phosphorylation step, to obtain the 5’-triphosphorylated oligonucleotide. The triphosphorylation was performed with a machine adapted version of Goldeck *et al.* on an OP-10 syntesizer.^[2]^**

Cleavage for oligonucleotides **Ȧ_6_, Ȧ_7_ and Ȧ_6,7_** was carried out using 2 mL AMA (1:1 solution of 40% aqueous methylamine and concentrated aqueous ammonia, freshly prepared) and two syringes. The oligonucleotides were brought in contact with AMA for 30 minutes. Afterwards, solution was transferred to a reaction vial and the remaining solid-support was rinsed with AMA. For deprotection, the combined solution was now heated to 65 °C for 10 min and then cooled to room temperature. The solvent was removed at 4 °C using a vacuum concentrator. To remove the 2’-TBDMS-protecting groups, **Ȧ_6_, Ȧ_7_ and Ȧ_6,7_** were redissolved in 115 μL DMSO. 60 μL Et_3_N and 75 μL Et_3_N*3HF were added and the solution was incubated at 65 °C for 2.5 h. After cooling the reaction mixtures to room temperature, 25 μL 3 M NaOAc in water and 1 mL ethanol were added and the oligonucleotide was precipitated at -80 °C for 30 min and consequently pelleted by centrifugation for 30 min at 4 °C. The precipitation was repeated 3 times in total and the crude oligonucleotides were then purified by RP-HPLC.

**Table S1.** Overview of chemically synthesized oligonucleotides.

|  | **Sequence ( 5’ 🡪 3’)** |
| --- | --- |
| **Ṡ_1_** | ppp **G**AC GCU G_F_AC CCU GAA_Me_ GUU CAU C*U_F_*U |
| **Ȧ_6_** | p A*A*G AUG AAC UUC AGG GUC **A**GC GUC |
| **Ȧ_7_** | p A*A*G AUG AAC UUC AGG GU**C** AGC GUC |
| **Ȧ_6,7_** | p A*A*G AUG AAC UUC AGG GU**C** **A**GC GUC |

**G** = 2’-PPG-G, **A** = 2’-PPG-A, **C** = 2’-PPG-C, U_F_ = 2’-F-U, A_Me_ = 2’-OMe-A, G_F_ = 2’-F-G, p = phosphate, * = PTOs

2.2 Purification

**RP-HPLC**

Purification of the crude oligonucleotides **Ȧ_6_, Ȧ_7_ and Ȧ_6,7_** was performed on an *Agilent 1200* *series* instrument using a *XBridge Peptide BEH C18 OBD Prep Column* (300 Å, 5 μm, 10x250 mm) from *Waters*. Elution was carried out with a solvent-gradient containing solvent A: methanol and solvent B: 400 mM HFIP (hexafluoroisopropanol), 16.3 mM Et_3_N, pH 7.9, with 4.0 mL/min at 60 °C (Table S2).

**Table S2.** Gradient used for RP-HPLC purification of **Ȧ**_6_, **Ȧ**_7_ and **Ȧ**_6,7_.

| **Time / min** | **%A** |
| --- | --- |
| **0** | 5 |
| **2** | 5 |
| **25** | 40 |
| **29** | 100 |

2.3 Mass spectrometry

**High-resolution mass spectrometry**

Purity and identity of **Ȧ_6_, Ȧ_7_ and Ȧ_6,7_** oligonucleotides was confirmed by analytical RP-HPLC and LC-MS. RP-HPLC was performed on an *Agilent 1200 series* instrument using a *XBridge Peptide BEH C18* column (300 Å, 3.5 μm, 4.6x250 mm) from *Waters*. Elution was carried out with a solvent-gradient containing solvent A: methanol and solvent B: 400 mM HFIP (hexafluoroisopropanol), 16.3 mM Et_3_N, pH 7.9, with 0.7 mL/min at 60 °C (Table S2). Mass spectra of not triphosphorylated oligonucleotides was obtained on a *Bruker micrOTOF-QII* device.

**Low-resolution mass spectrometry**

**Purity and identity of Ṡ_1_** and additionally for **Ȧ_6_, Ȧ_7_ and Ȧ_6,7_** was confirmed by **LC-MS analysis which was performed using an ACQUITY-UPLC system (*Waters*) coupled to an *Xevo TQ-S Quadrupole* (*Waters*) equipped with an electrospray source operating in negative ionization mode. All samples were chromatographed on a ACQUITY UPLC BEH C18 column (2.1 x 50 mm; 1.8 μm particle size) at 60 °C column temperature. Separation of the analytes was achieved using a gradient of 16.6 mM triethylamine (TEA), 100 mM hexafluoroisoproanol (HFIP) and 10% methanol as eluent A and 16.6 mM TEA, 100mM HFIP and 95% MeOH as eluent B with a flow rate of 0.3 mL/min. A segmented gradient was run according to table S3. Samples were prepared in 200 mM TEAA and 20 mM EDTA.**

**Table S3.** Gradient used for LC-MS analysis of **Ṡ_1._**

| **Time / min** | **%A** | **%B** |
| --- | --- | --- |
| **0** | 100 | 0 |
| **1.50** | 100 | 0 |
| **5** | 93 | 7 |
| **11.25** | 85 | 15 |
| **15.75** | 60 | 40 |
| **16** | 0 | 100 |
|  |  |  |

**Table S4.** Calculated and found masses for all synthesized oligonucleotides.

|  | **Calculated Mass** | **Found Mass** |
| --- | --- | --- |
| **Ṡ_1_** | 8053.9208 | 8054 |
| **Ȧ_6_** | 8002.0479 | 8002.8835 |
| **Ȧ_7_** | 8002.0479 | 8004.0598 |
| **Ȧ_6,7_** | 8181.1061 | 8182.1421 |

**
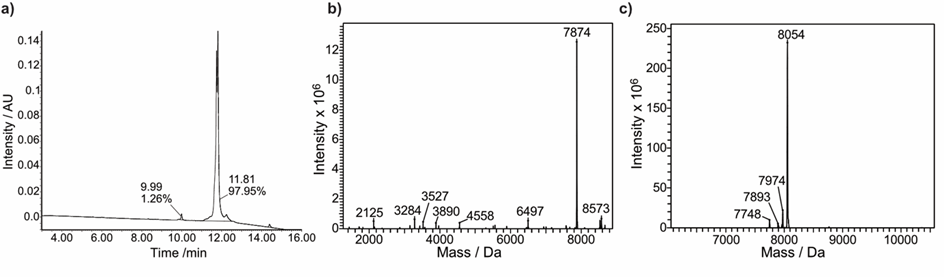
Ṡ_1_**


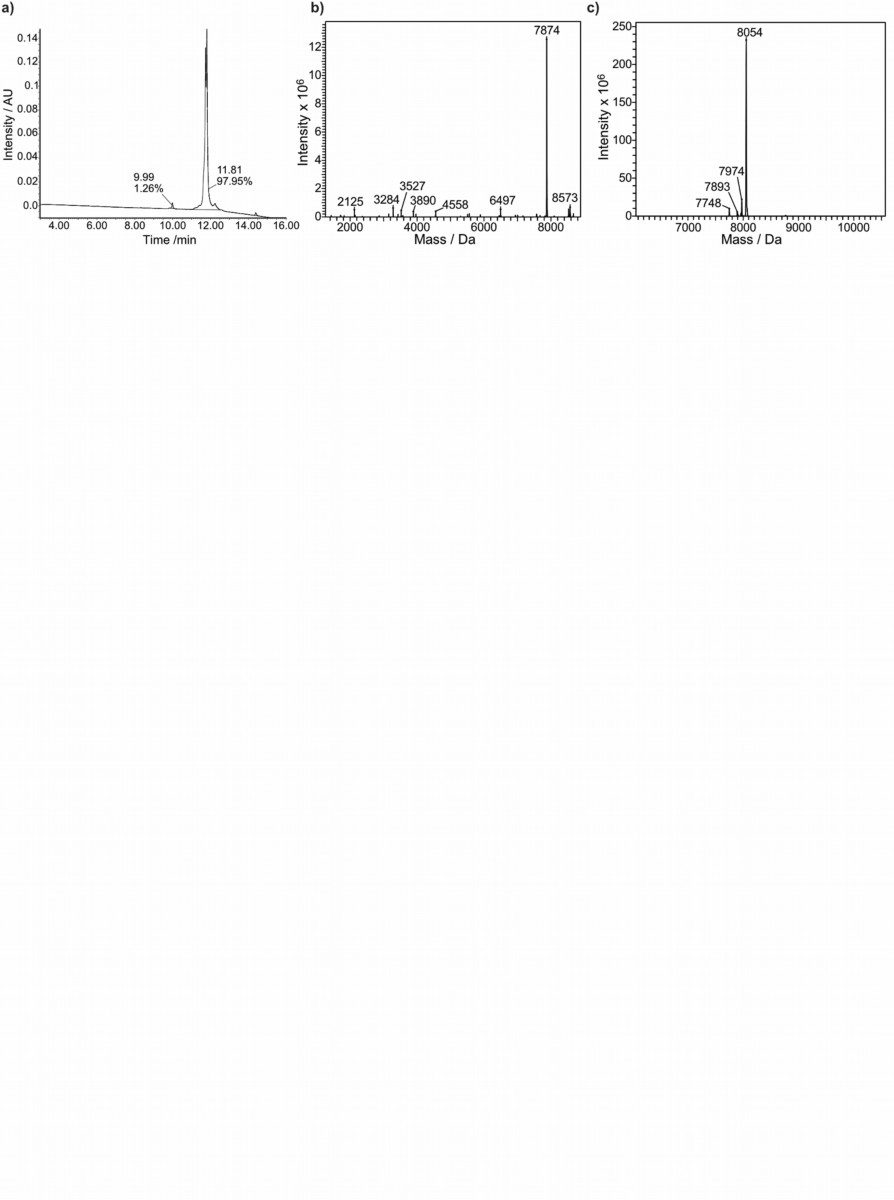


**Figure S1:** LC-MS Data from **Ṡ_1_**. **a)** LC UV chromatogram with percentage of peak AU **b)** deconvoluted mass from peak at 9.99 min, mass equals RNA mass after PPG cleavage **c)** deconvoluted mass from peak at 11.81 min, mass equals RNA mass with PPG

**Ȧ_6_**

**a) b)**


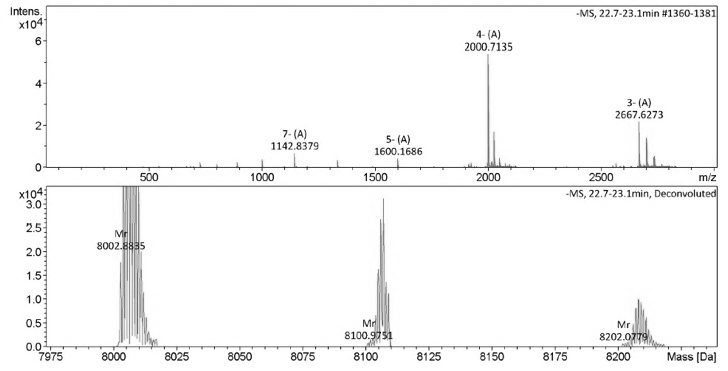

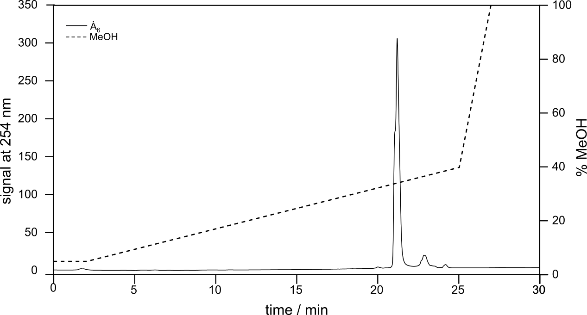


**Figure S2:** LC-HRMS Data from **Ȧ_6_**. **a)** analytical UV RP-HPLC chromatogram **b)** top: mass spectrum of **Ȧ_6,_** bottom: deconvoluted mass spectrum of **Ȧ_6_**.


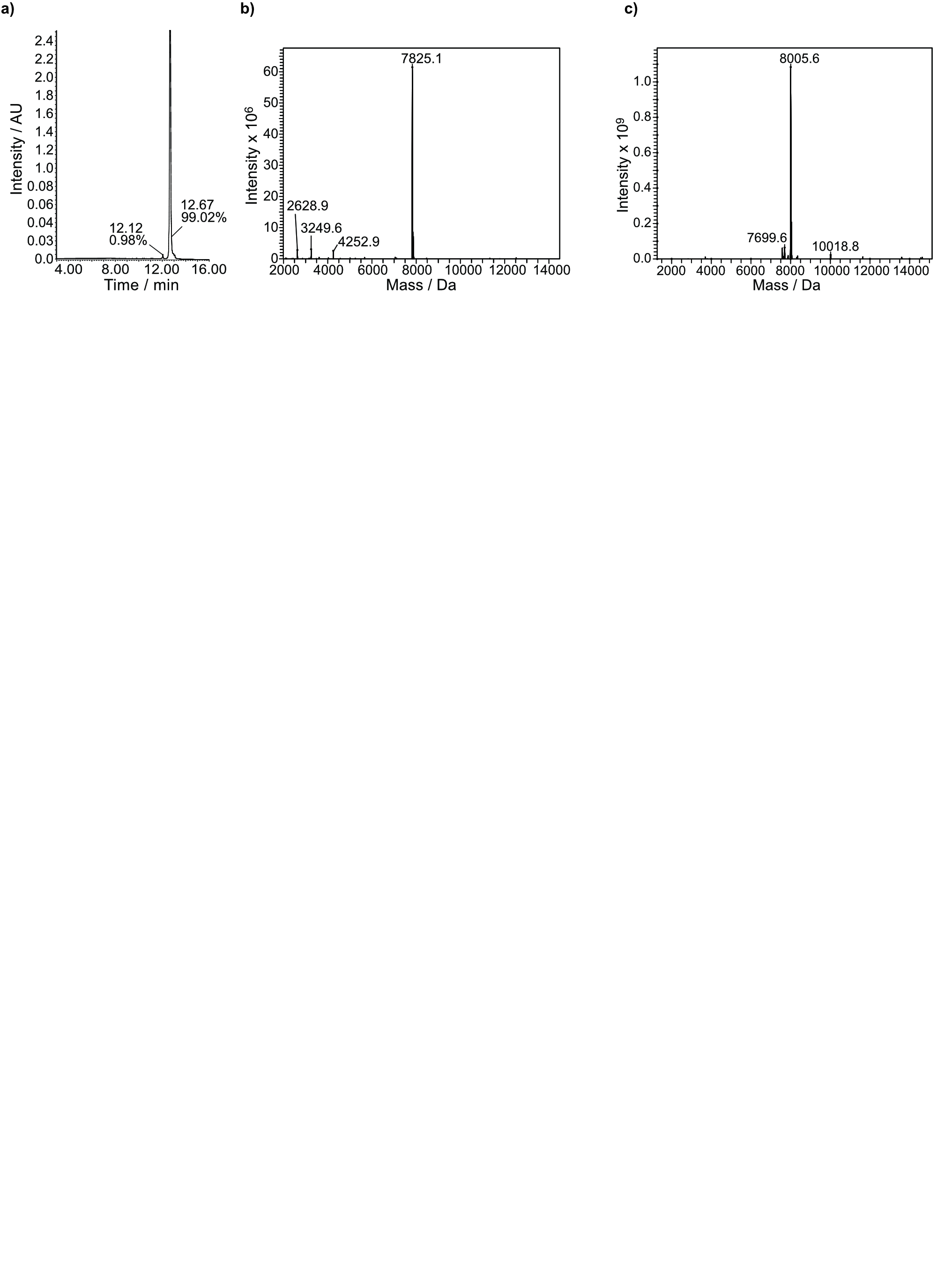


**Figure S3:** LC-MS Data from **Ȧ_6_**. **a)** LC UV chromatogram with percentage of peak AU **b)** deconvoluted mass from peak at 12.12 min, mass equals RNA mass after PPG cleavage **c)** deconvoluted mass from peak at 12.67 min, mass equals RNA mass with PPG.

**Ȧ_7_**

**a) b)**


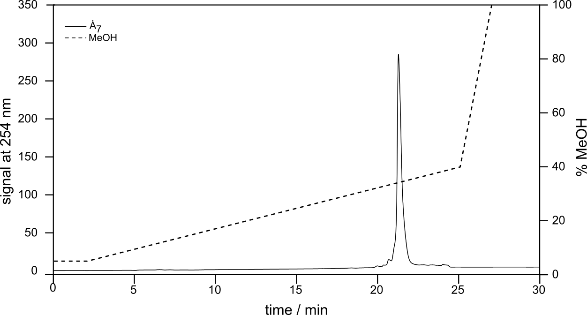

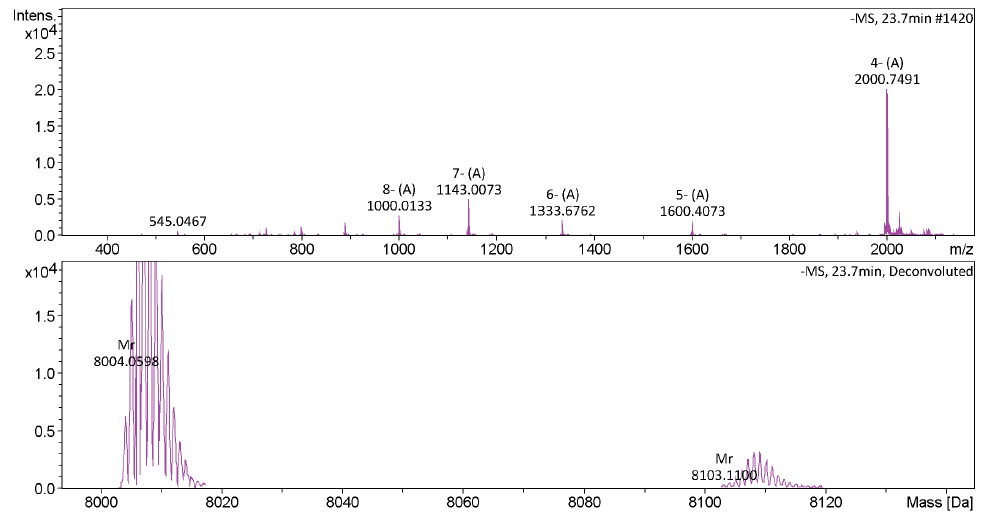


**Figure S4:** LC-HRMS Data from **Ȧ_7_**. **a)** analytical UV RP-HPLC chromatogram **b)** top: mass spectrum of **Ȧ_7,_** bottom: deconvoluted mass spectrum of **Ȧ_7_**.


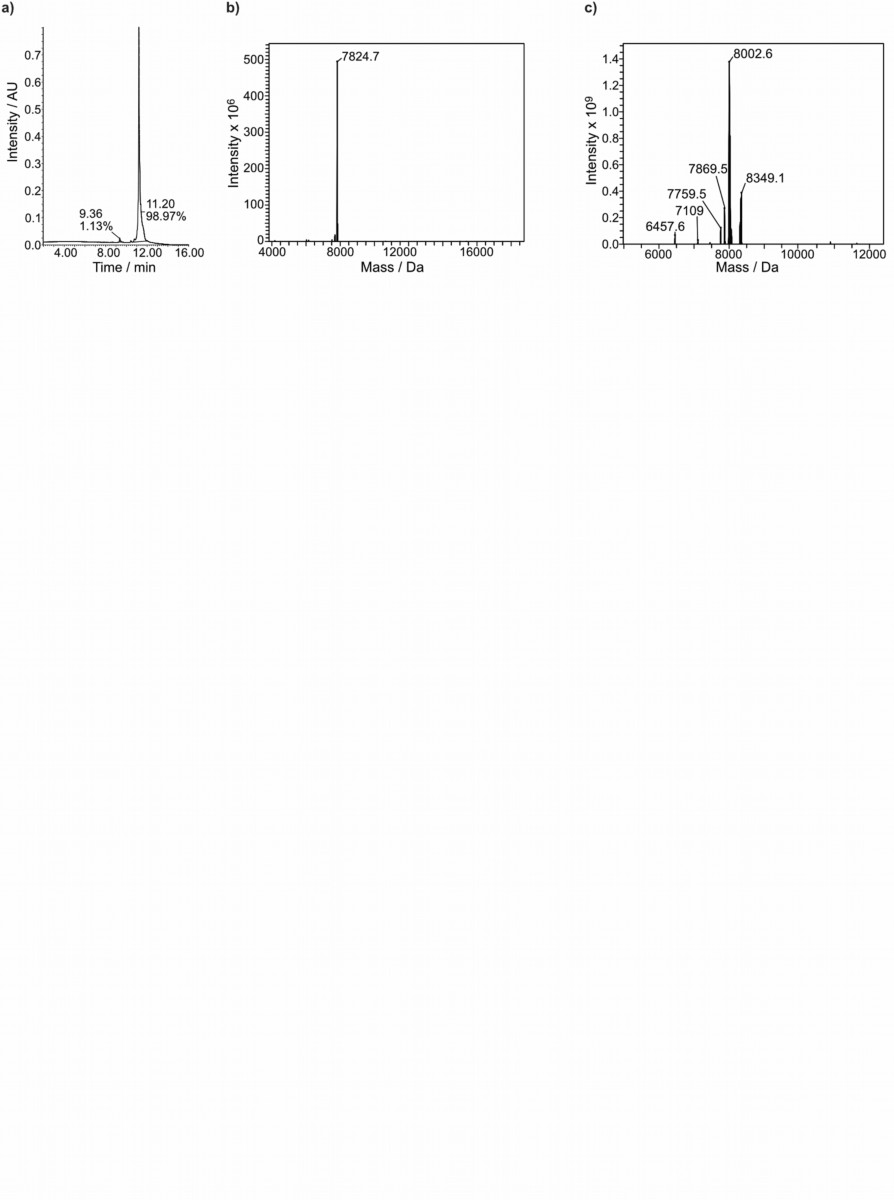


**Figure S5:** LC-MS Data from **Ȧ_7_**. **a)** LC UV chromatogram with percentage of peak AU **b)** deconvoluted mass from peak at 9.36 min, mass equals RNA mass after PPG cleavage **c)** deconvoluted mass from peak at 11.20 min, mass equals RNA mass with PPG.

**Ȧ_6,7_**

**a) b)**


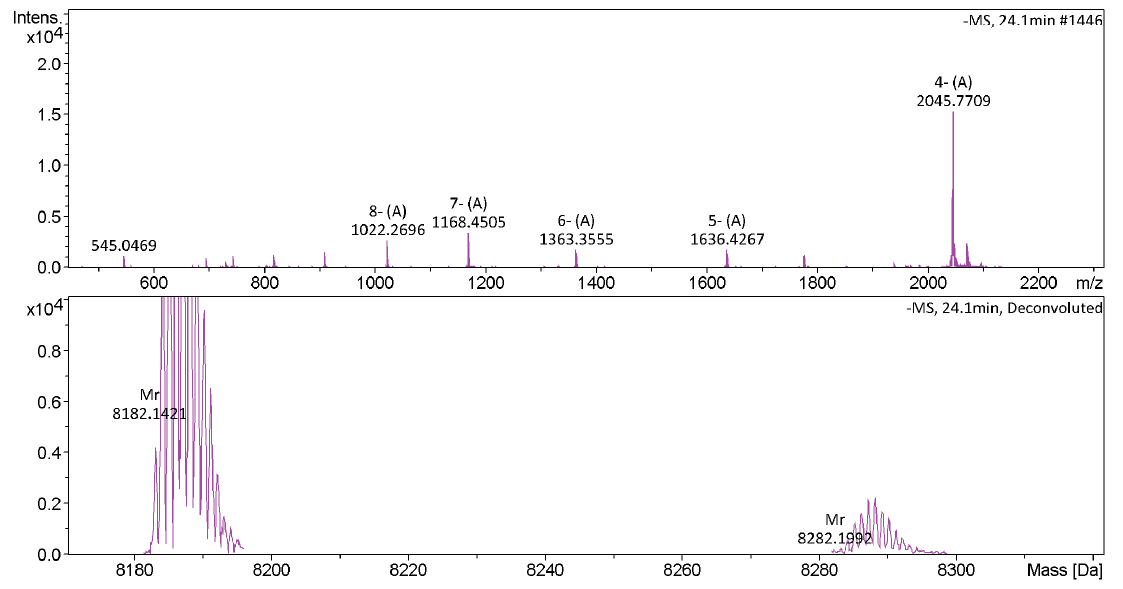

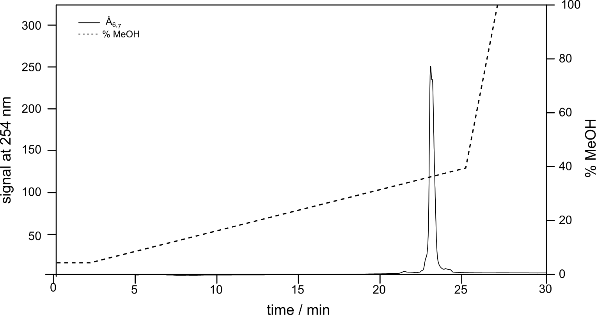


**Figure S6:** LC-HRMS Data from **Ȧ_6,7_**. **a)** analytical UV RP-HPLC chromatogram **b)** top: mass spectrum of **Ȧ_6,7,_** bottom: deconvoluted mass spectrum of **Ȧ_6,7_**.


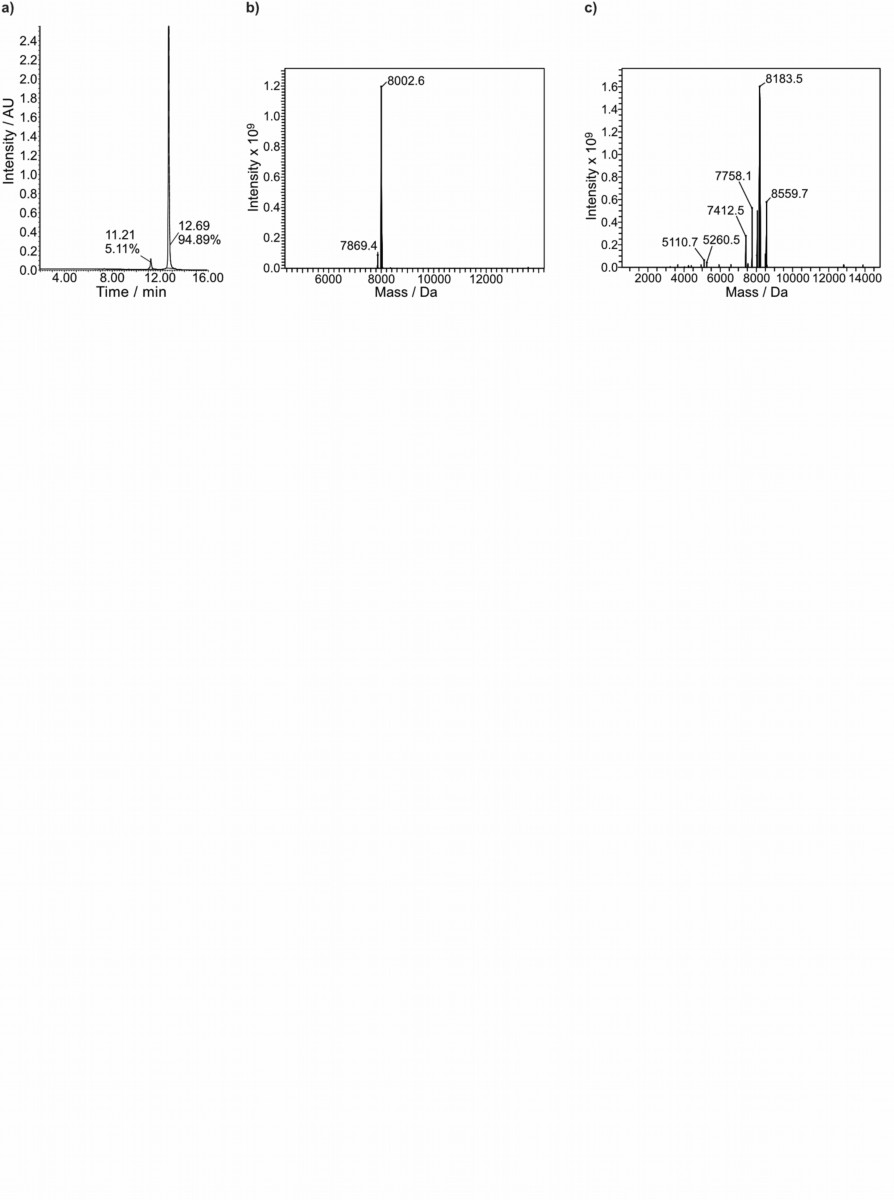


**Figure S7:** LC-MS Data from **Ȧ_6,7_**. **a)** LC UV chromatogram with percentage of peak AU **b)** deconvoluted mass from peak at 11.21 min, mass equals RNA mass after one PPG cleavage **c)** deconvoluted mass from peak at 12.69 min, mass equals RNA mass with PPG

2.4 Experimental irradiation of Ȧ_6,7_

To demonstrate the functionality of the photolabile protecting group, the anti-sense strand **Ȧ_6,7_** was irradiated exemplary. A 1 μM solution of the single stranded oligonucleotide in DEPC-H_2_O was irradiated for 10 minutes. The irradiation was carried out using a 365 nm LED (M365L2, *Thorlabs*) at a power of P = 2 mW. The irradiated sample was analyzed by RP-HPLC and mass spectrometry to determine the photolysis product (Scheme S1).


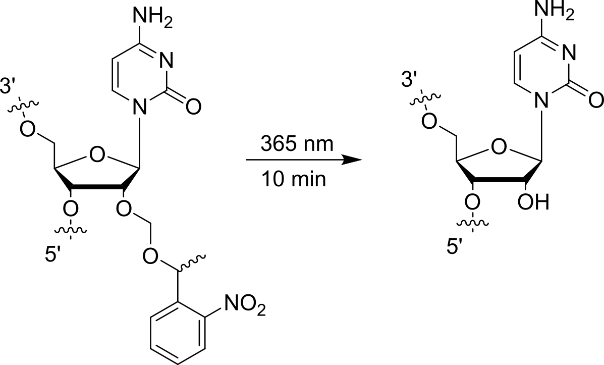


**Scheme S1:** Photolysis of a 2’-photolabile protected nucleotide.

RP-HPLC was performed on an *Agilent 1200 series* instrument using a *XBridge Peptide BEH C18* column (300 Å, 3.5 μm, 4.6x250 mm) from *Waters*. Elution was carried out with a solvent-gradient containing solvent A: methanol and solvent B: 400 mM HFIP (hexafluoroisopropanol), 16.3 mM Et_3_N, pH 7.9, with 0.7 mL/min at 60 °C (Table S2). Mass spectra of the new formed products was obtained on a *Thermo Fisher Orbitrap Exploris 120* device.

**Table S5:** Calculated and found masses for irradiated **Ȧ_6,7_**.

|  | **Calculated Mass** | **Found Mass** |
| --- | --- | --- |
| **Ȧ_6,7_** with only 1 photolabile protecting group | 8002.0479 | 8005.8550 |
| **Ȧ_6,7_** without any photolabile protecting group | 7822.9897 | 7823.7993 |


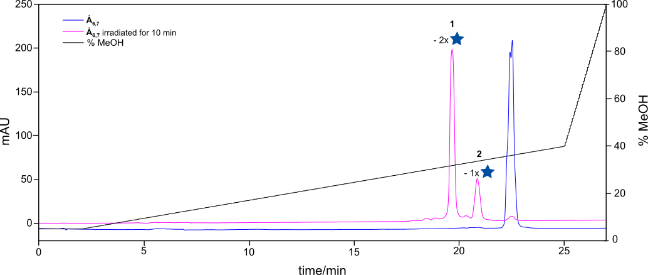


**Figure S8:** RP-HPLC chromatogram before (blue) and after (pink) irradiation of **Ȧ_6,7_**. *XBridgePeptide* BEH C18 Column, HFIP-Buffer (400 mM HFIP, 16.3 mM TEA, pH=8), c(**Ȧ_6,7_** ) = 1 µM, λ_irr_ = 365 nm, 10 min, P = 2 mW.

**1: 2:**


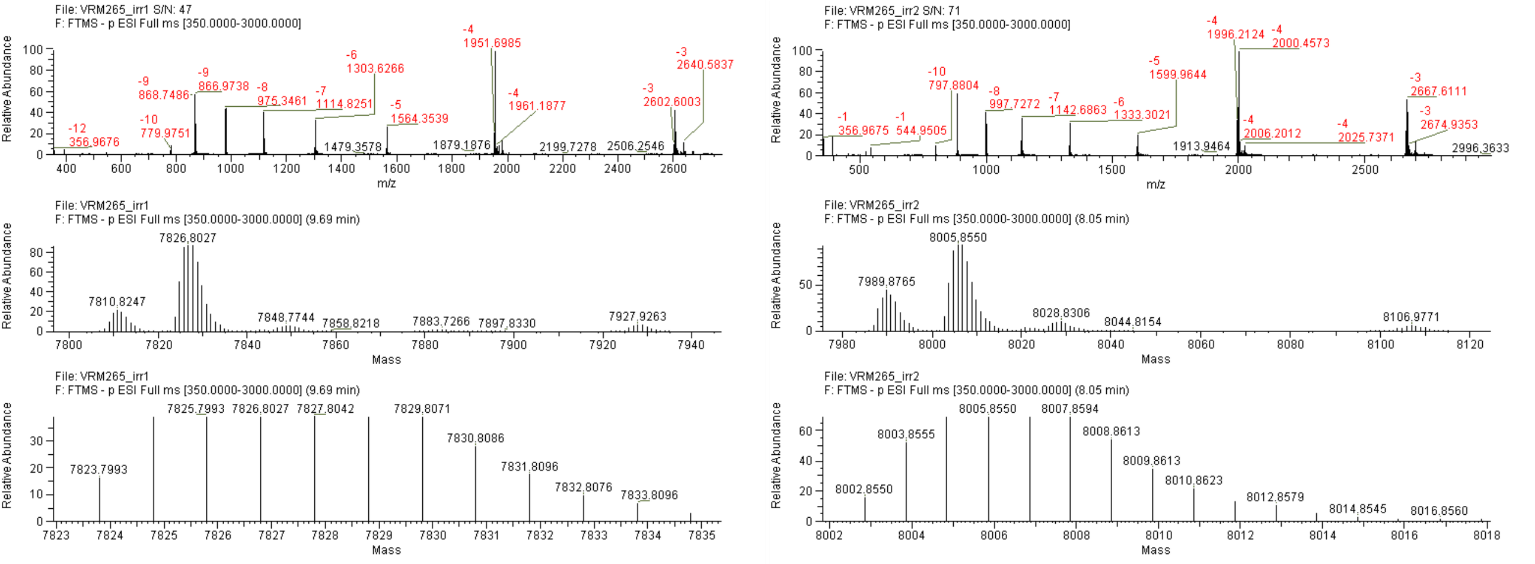


**Figure S9:** **1:** top: mass spectrum of peak 1, shown in RP-HPLC chromatogram in Figure S1, middle: deconvoluted mass spectrum of peak 1, bottom: zoomed in deconvulted mass spectrum of peak 1 1. **2:** top: mass spectrum of peak 2, shown in RP-HPLC chromatogram in Figure S1 middle: deconvoluted mass spectrum of peak 2, bottom: zoomed in deconvulted mass spectrum of peak 2.

3. Cell experiments to measure RIG-I activity and RIG-I induced apoptosis

3.1 Materials and Methods

**Ethics statement**

The studies involving human peripheral blood mononuclear cells (PBMCs) were approved by the local ethics committee (Ethics Committee of the University Hospital Bonn, ID-number 516/20) according to the ICH-GCP guidelines. Written informed consent was provided by voluntary blood donors. The experiments involving patient material were approved by the Ethics Committee of the University Hospital Bonn (EK 219/17). Informed, written consent was obtained from each patient included in the study.

**Human primary cells and cell lines**

Peripheral blood mononuclear cells (PBMCs) were isolated by Ficoll-Hypaque density gradient centrifugation from whole blood of healthy volunteers and from buffy coats using Ficoll^®^ Paque Plus (Sigma-Aldrich). PBMCs were plated in RPMI 1640 full medium (supplemented with 10% fetal calf serum (FCS), 100 µg/ml penicillin, 100 μg/ml streptomycin, 1 mM sodium pyruvate and 0,1 mM MEM non-essential amino acids (all from Thermo Fisher Scientific)) and incubated in a humidified incubator at 37°C and 5% CO_2_ at a density of 4x10^6^ cells/mL.

THP1 dual (Invivogen) were cultured in RPMI 1640 full medium in a humidified incubator at 37°C and 5% CO2 and plated at a density of 8x10^5^ cells/mL in a 96 well plate.

HeLa (CCL-2; ATCC), A375 and A549 cells were cultured in DMEM high glucose full medium (supplemented with 10% fetal calf serum (FCS), 100 µg/ml penicillin, 100 μg/ml streptomycin, 1 mM sodium pyruvate and 0,1 mM MEM non-essential amino acids (all from Thermo Fisher Scientific)), seeded one day before transfection at a density of 2x10^5^ cells/mL, if not else specified, and incubated in a humidified incubator at 37°C and 5% CO2. A375 cells were kindly provided by Michael Hölzel (University Hospital Bonn, Germany) and A549 cells were kindly provided by Christine Goffinet (Charité Berlin, Germany).

Primary ccRCC (clear cell renal cell carcinoma) cells were isolated as previously described^[3]^ and cultivated in DMEM/F12 medium (supplemented with 5% FCS (Thermo Fisher Scientific), 1% penicillin/streptomycin (Thermo Fisher Scientific), 10 ng/mL hrEGF (R&D Systems), 10 ng/mL FGF-basic (PeproTech), 4 µg/ml Heparin (Thermo Scientific Chemicals), 1 × B27-supplement without vitamin A (Thermo Fisher Scientific), 1 × Lipid Mixture 1 (Sigma-Aldrich), 1-mM *N*-Acetyl-Cysteine (Thermo Fisher Scientific), 1 x GlutaMAX (Thermo Fisher Scientific), 1 × MEM non-essential amino acids (Thermo Fisher Scientific) and 10-mM HEPES (GE Healthcare, UK) in a humidified incubator at 37°C and 5% CO_2_. One day before transfection ccRCC cells were seeded in 96-well plates at a density of 2x10^5^ cells/mL.

Cell lines were verified to be mycoplasma negative by using a mycoplasma-specific PCR approach at regular intervals.

For experiments involving irradiation by light, cells were seeded in black 96-well plates with their respective culture medium without phenolred.

**Cell stimulation**

If not stated otherwise, transfection was performed with the indicated concentrations of oligonucleotides using Lipofectamine2000® (Invitrogen) according to the instructions. Briefly, the oligonucleotides and Lipofectamine 2000 dilutions were prepared in OptiMEM (Thermo Fisher Scientific) and incubated for 5 min. Afterward, the RNA/OptiMEM and Lipofectamin/OptiMEM solutions were mixed in equal volume and incubated for additional 20 min at RT. 25 µL of the mixture was added to each well resulting in the indicated concentration.

**Elisa**

Secretion of IFN-α (Thermo Fisher Scientific) and CXCL10 (BD Biosciences) was quantified in cell-free supernatants by ELISA according to the manufacturer's instructions.

**Lucia luciferase assay**

Activity of the THP1 dual ISRE reporter (interferon-stimulated response elements) was measured by quantifying the Lucia luciferase reporter gene expression. Cell-free supernatants were mixed in a 1:1 ratio with Luciferase Buffer (100 mM Tris pH 7,4, 300 mM sodium ascorbate) including 1 µg/mL Coelenterazine substrate. The luminescence was measured using an EnVision 2104 Multilabel Reader (PerkinElmer).

**A549 IFNβ-eGFP reporter cells**

A549 wildtype cells were electroporated (1230 V, 30 ms, 2 pulses, 100 µL tip) with an Invitrogen™ Neon™ Transfection System with sgRNA (IFNβ-locus)-Cas9 expressing and IFNβ-promoter-eGFP expressing plasmids. Single cell clones were obtained by limiting dilution. The single cell clone with the best signal-noise ratio was used for shown experiments. Depending on the experiment, A549 IFNβ-eGFP cells were seeded the day before transfection either at a density of 2x10^5^ cells/mL for eGFP quantification or 5x10^4^ cell/mL for microscopy images. Transfection was performed as described above. After 48 hours of incubation cells were used for fluorescence readout. Quantification of eGFP positive cells was performed on Cytation3 multimode reader (BioTek) and analyzed with Gen5 software (Agilent). Bright field images were used for quantification of total cell number. EGFP signal (λex =469 nm, λem = 525 nm) was used for quantification of eGFP positive cells. The percentage of eGFP positive cells was obtained by normalization of eGFP positive cell count to total cell count per well. For each experiment technical duplicates were analyzed.

A Leica DMi 8 microscope with fluorescence camera (Leica-DFC9000GT) was used to image the treated cells with an x40 objective lens (HC PL APO 40x/0.95 DRY) or a x20 objective lens (HC PL Fluotar L 20x/0.40 DRY). Images were captured at bright field for total cell image and at a green channel for eGFP fluorescence (λex = 475 nm, λem = 535 nm). Images were edited to same brightness and contrast levels using Fiji software. For spatial controlled irradiation, cells were plated in a 6 well plate and light was confined by cardboard with a 0,3 µm hole.

**FACS staining and analysis**

After 72 hours of incubation cells were harvested and washed with 100 µL PBS (phosphate buffered saline, Thermo Fisher Scientific/Gibco). Afterwards, cells were incubated with Annexin V-Alexa 647 (diluted 1:30; BioLegend) in Annexin binding buffer (10 mM HEPES, pH 7,4; 140 mM NaCl; 2,5 mM CaCl_2_) for 15 minutes of incubation at RT in the dark. Subsequently, cells were resuspended in 200 µL Annexin binding buffer, containing 1,25 µg/mL 7-AAD (7-Aminoactinomycin D, Enzo Life Sciences). FACS measurement was performed immediately after the staining process. Fluorescence intensities were measured with the Attune NxT Flow Cytometer (Thermo Fischer) and results analyzed using FlowJo software.

3.2 Irradiation of photolabile RNA-ligands in cells

The irradiation of the photolabile RNA ligands in cells was carried out using a self-made irradiation box, built by Martin Held shown in Figure S10. The box contains 96 LEDs, to irradiate each well on a 96-well plate. It is possible to irradiate the rows for different time lengths. The LEDs emit λ = 365 nm and P = 2 mW.


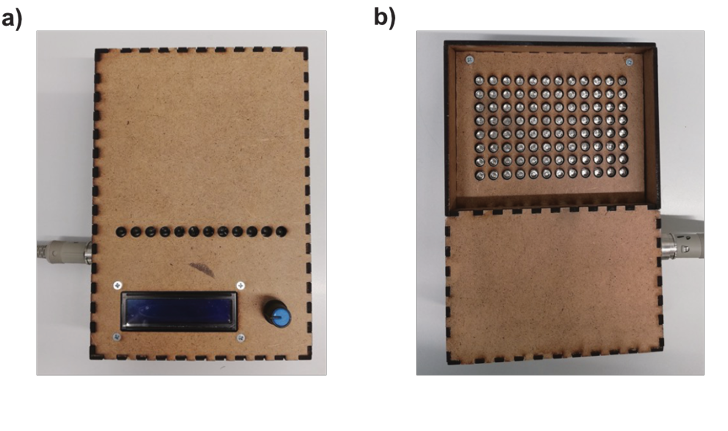


**Figure S10:** Irradiation box with 96 365 nm light emitting diodes. **a)** top view **b)** bottom view.


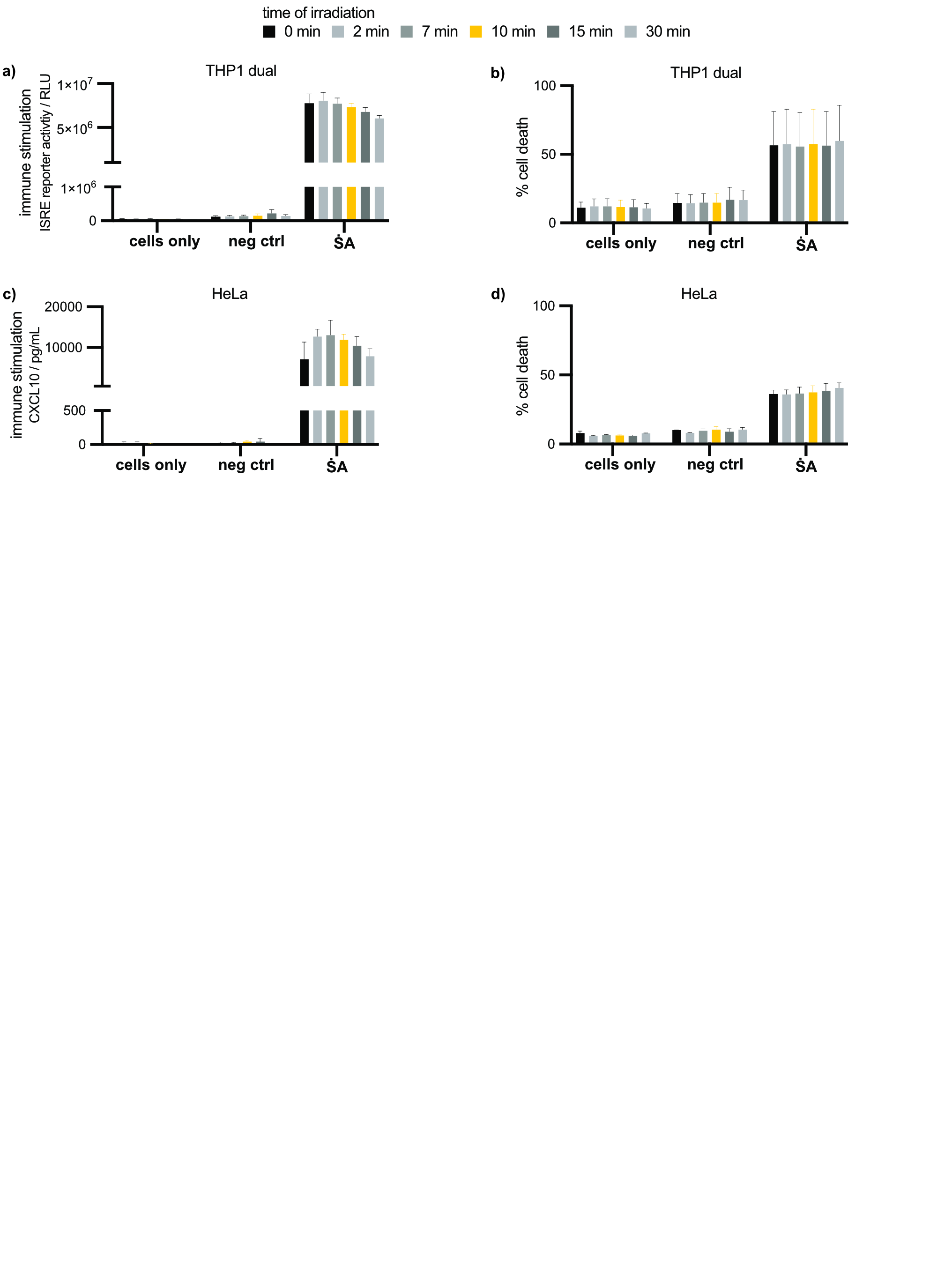
3.3 Titration of irradiation time

**Figure S11:** Effects of UV light irradiation on immune induction and cell death. **a), b)** THP1 dual cells or **c), d)** HeLa cells were transfected with 0,4 µg/mL of **ṠA, neg ctrl RNA** or nothing, followed by light irradiation for the indicated time (365 nm, 2 mW). Readouts were performed after 24 hours of incubation. **a)** Lucia luciferase readout for the ISRE reporter was performed in the supernatant. **b), d)** Percentage of dead cells was measured by FACS analysis of 7-AAD positive cells. **c)** CXCL-10 induction was measured by ELISA in the supernatant. **a-d)** Results show the mean ± SEM of at two independent experiments.

4. NMR-Spectra

***N^6^*-Benzoyl-5’-*O*-[bis(4-methoxyphenyl)phenylmethyl**]-**2′**-**O**-**[[(R,S)-1-(2-nitrophenyl)ethoxy]methyl**]**adenosine (2a)**

**^1^H (DMSO-*d*_6_):**


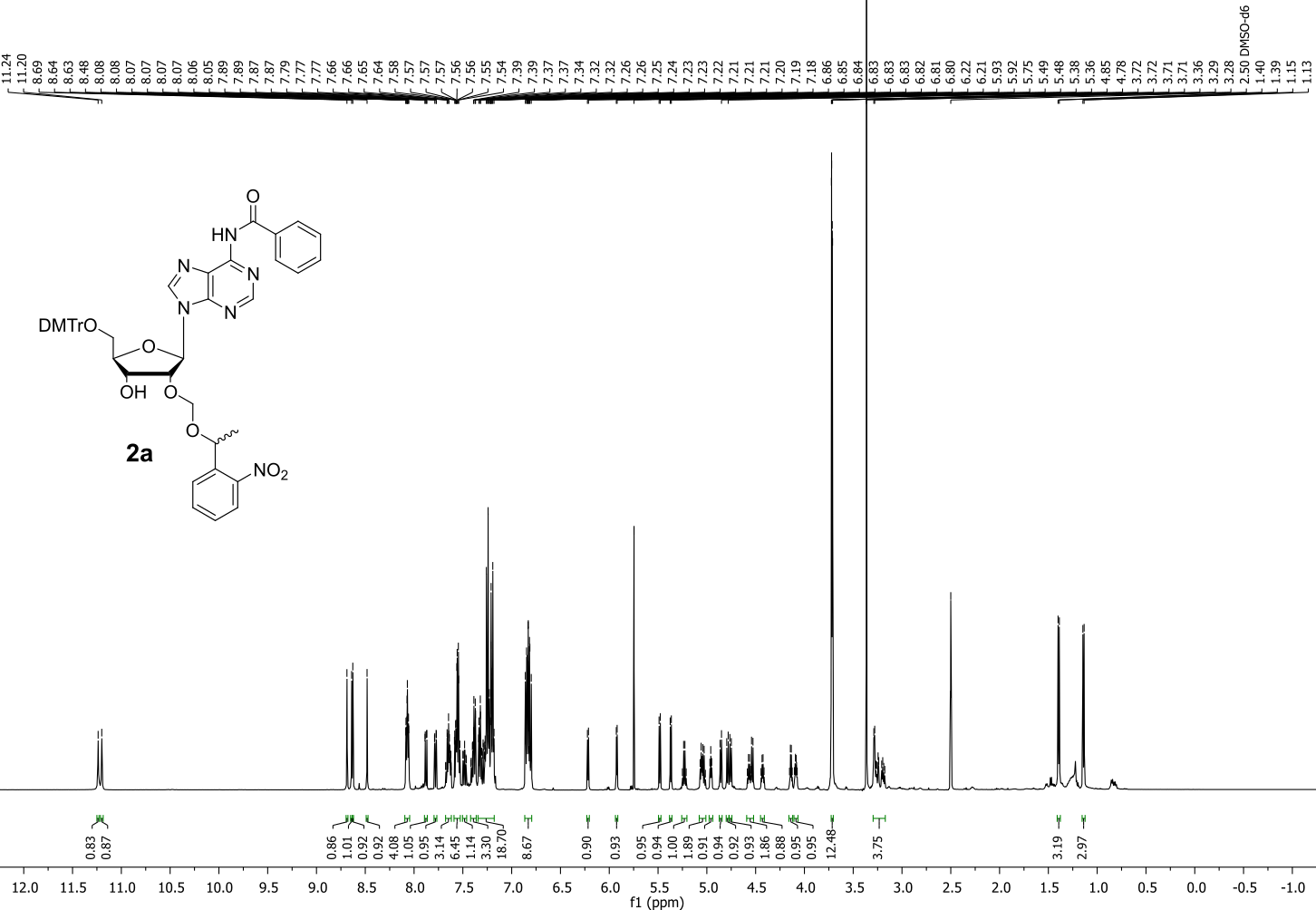


**^13^C{^1^H} (DMSO-*d*_6_):**


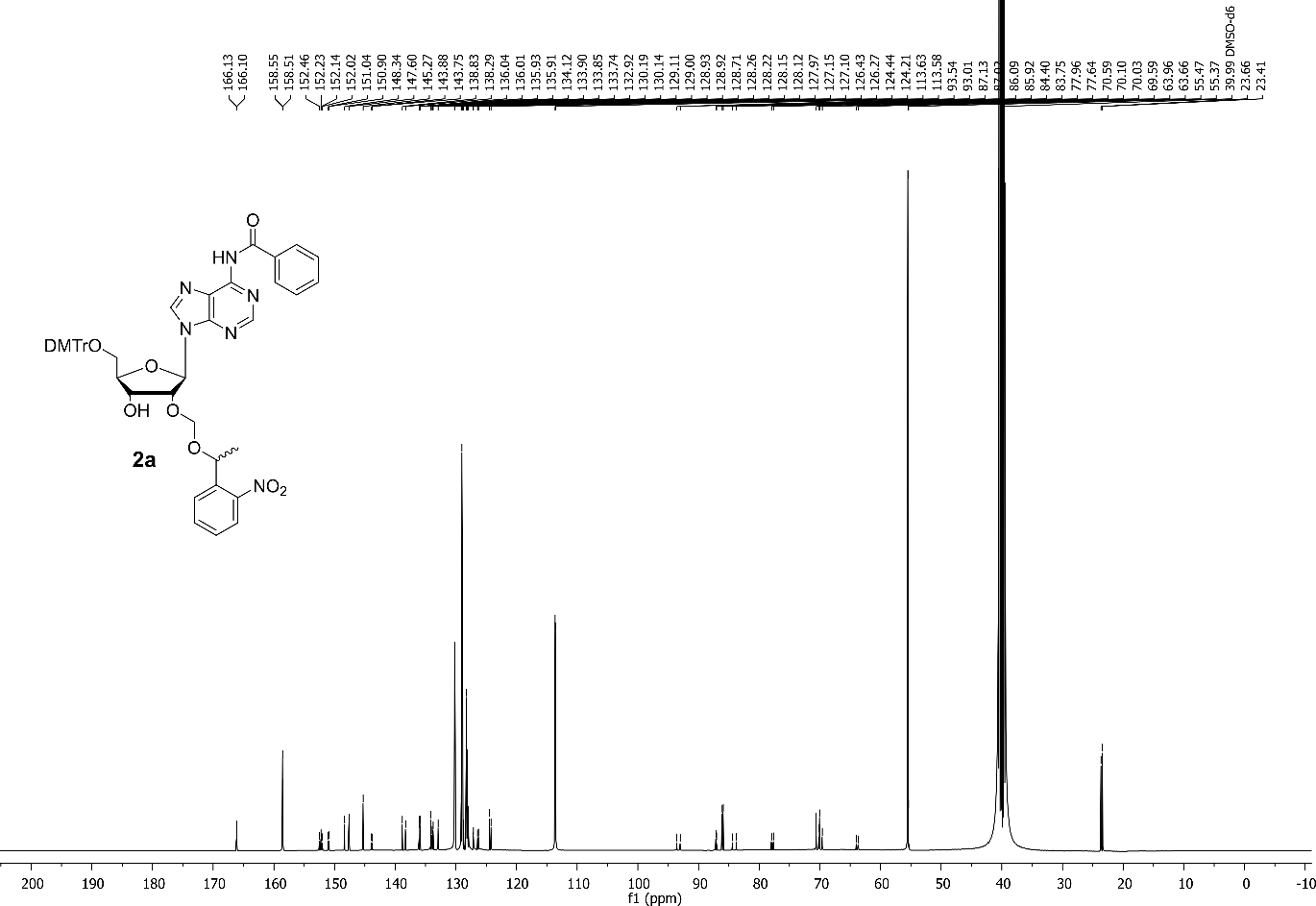


***N^6^*-Benzoyl-5’-*O*-[bis(4-methoxyphenyl)phenylmethyl]-2′-O-[[(R,S)-1-(2-nitrophenyl)ethoxy]methyl]adenosine-(2-cyanoethyl)diisopropylphosphoramidite (3a)**

**^1^H (DMSO-*d*_6_):**


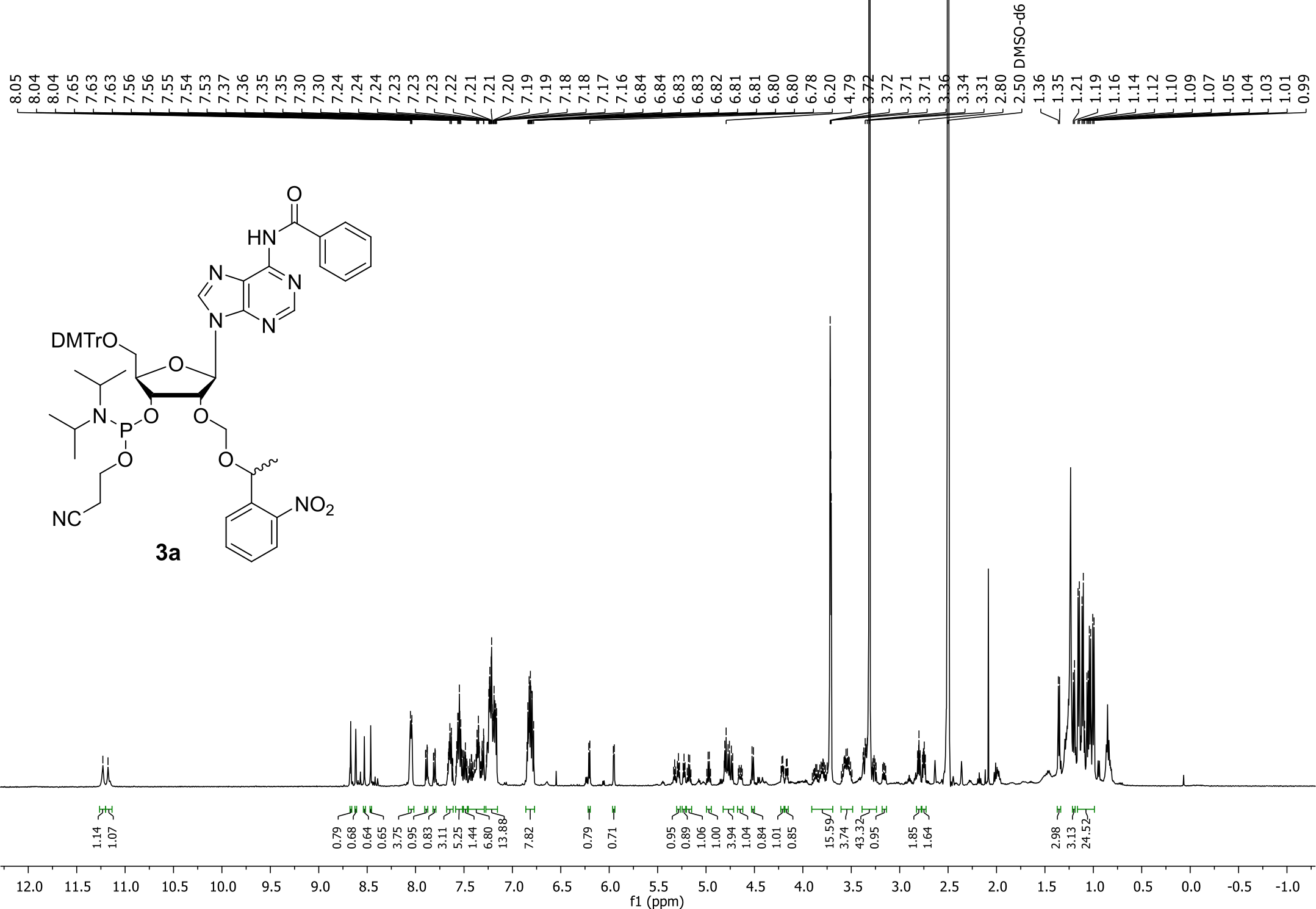


**^13^C{^1^H} (DMSO-*d*_6_):**


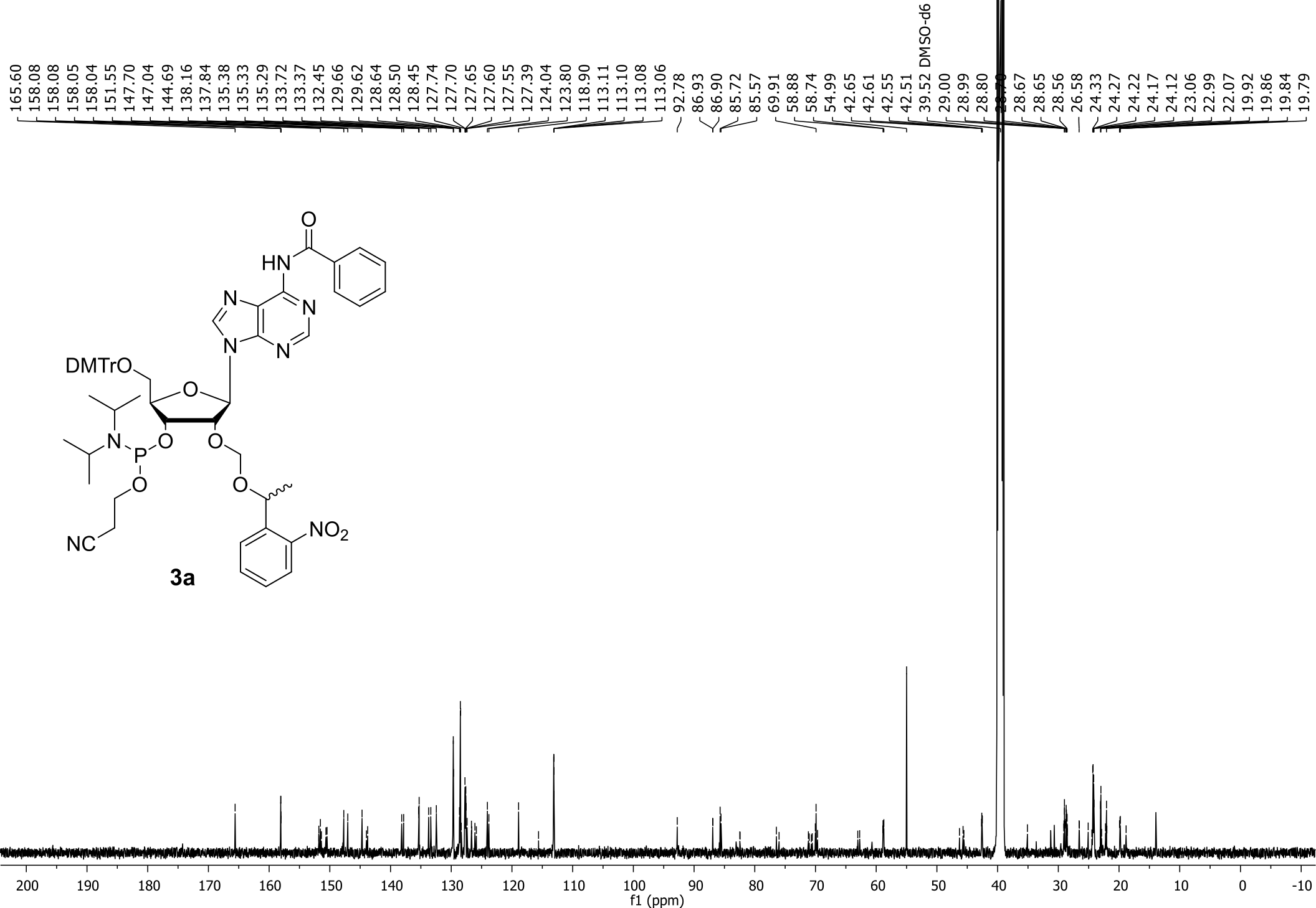


**^31^P{^1^H} (DMSO-*d*_6_):**


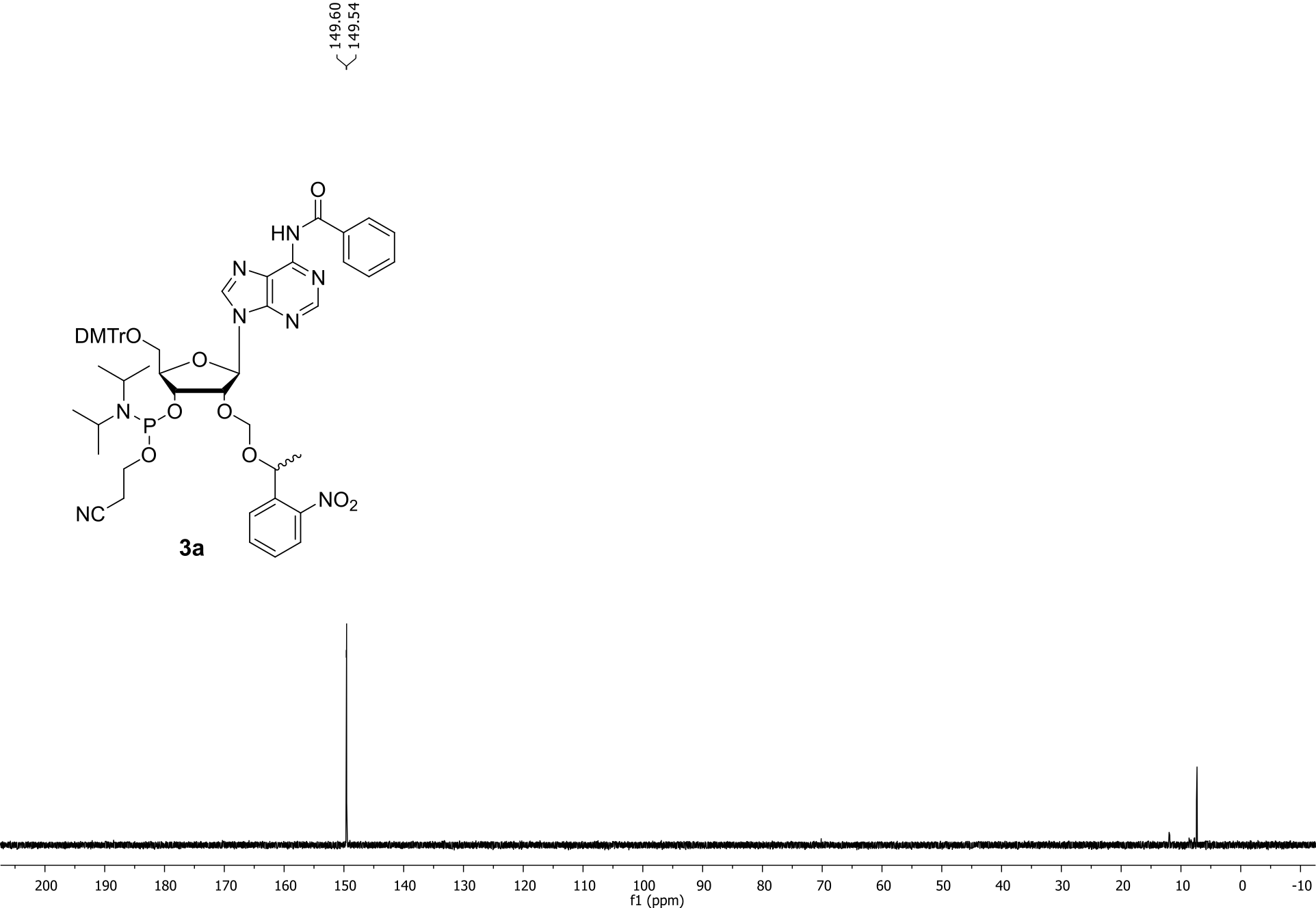


**5′-O-[Bis(4-methoxyphenyl)phenylmethyl]-N^2^-[(dimethylamino)methylene]**-2′-**O**-**[[(R,S)-1-(2-nitrophenyl)ethoxy]methyl]guanosine (2b)**

**^1^H (DMSO-*d*_6_):**


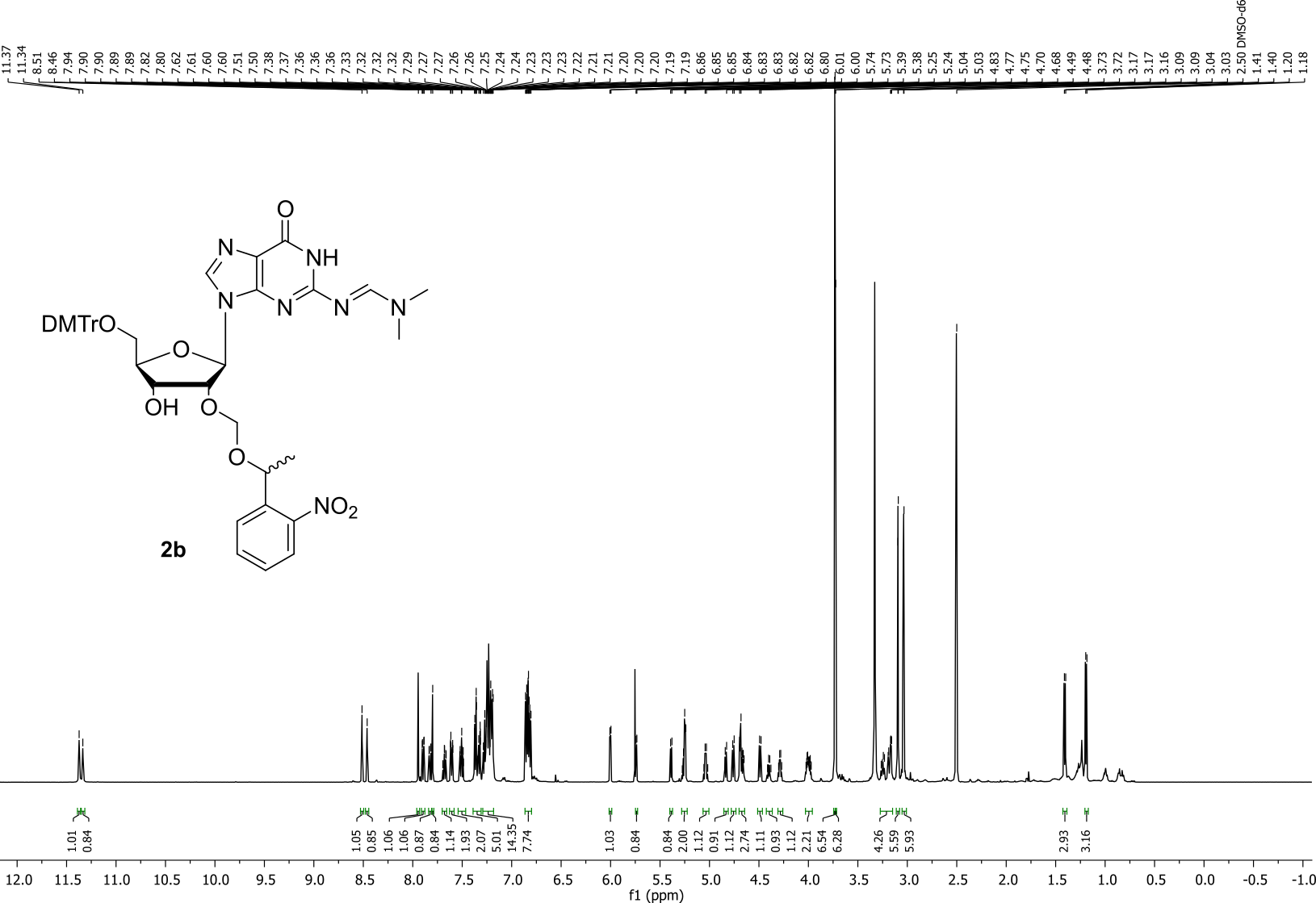

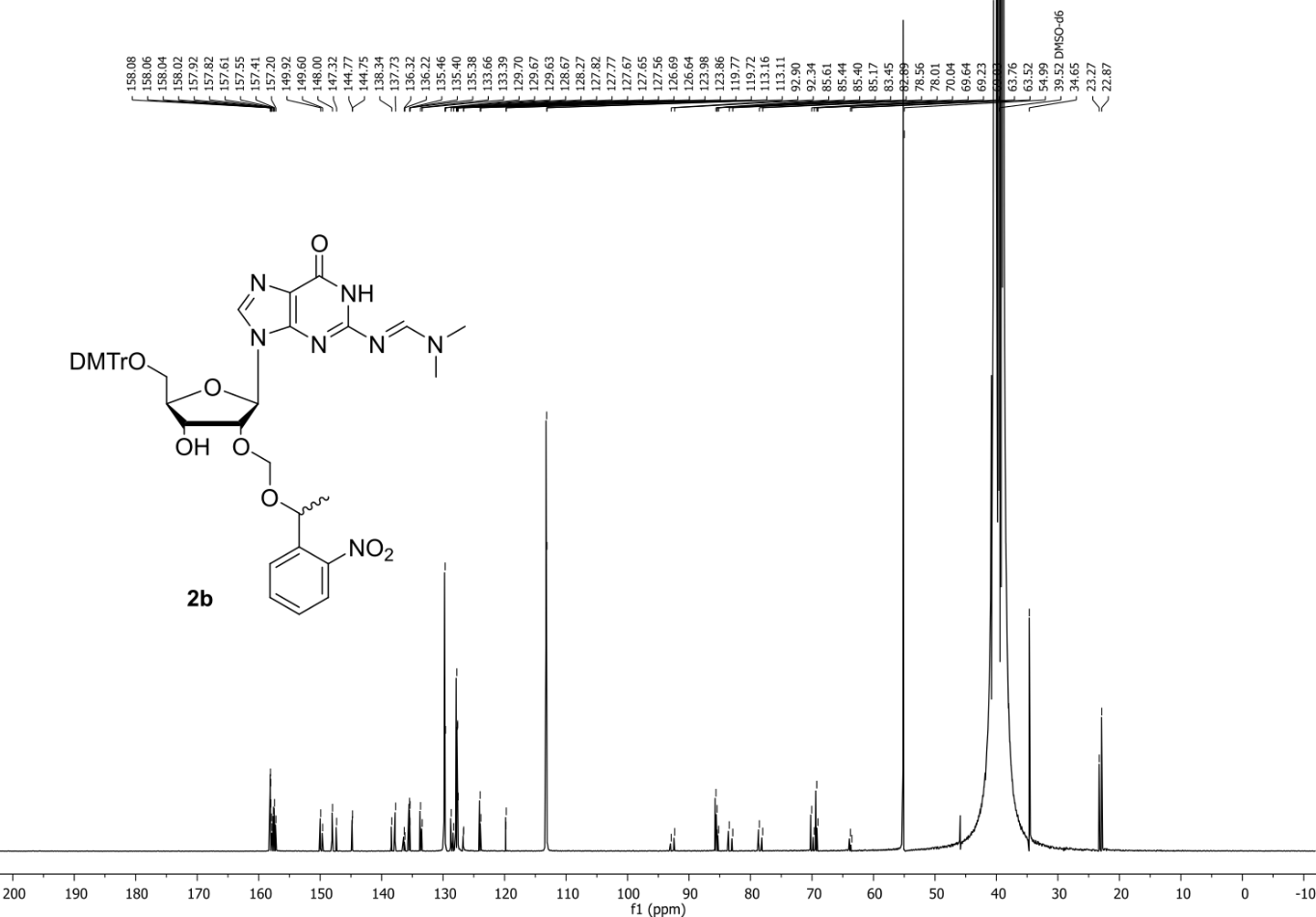


**^13^C{^1^H} (DMSO-*d6*):**

**5′-O-[Bis(4-methoxyphenyl)phenylmethyl]-N^2^-[(dimethylamino)methylene]**-2′-**O**-**[[(R,S**)**-1-(2-nitrophenyl)ethoxy]methyl]guanosine-(2-cyanoethyl)diisopropylphosphoramidite (3b)**

**^1^H (DMSO-*d*_6_):**


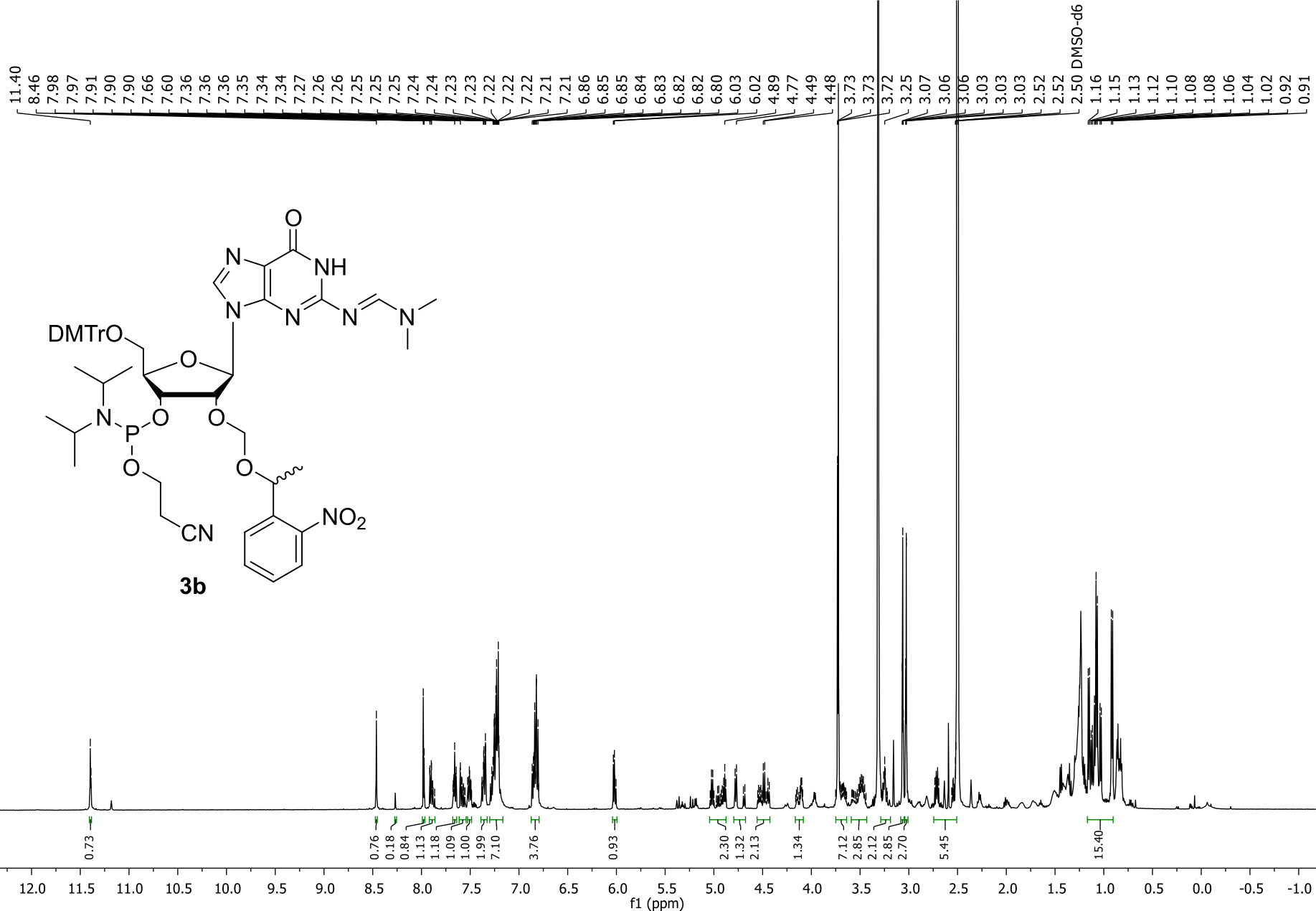


**^13^C{^1^H} (DMSO-*d*_6_):**


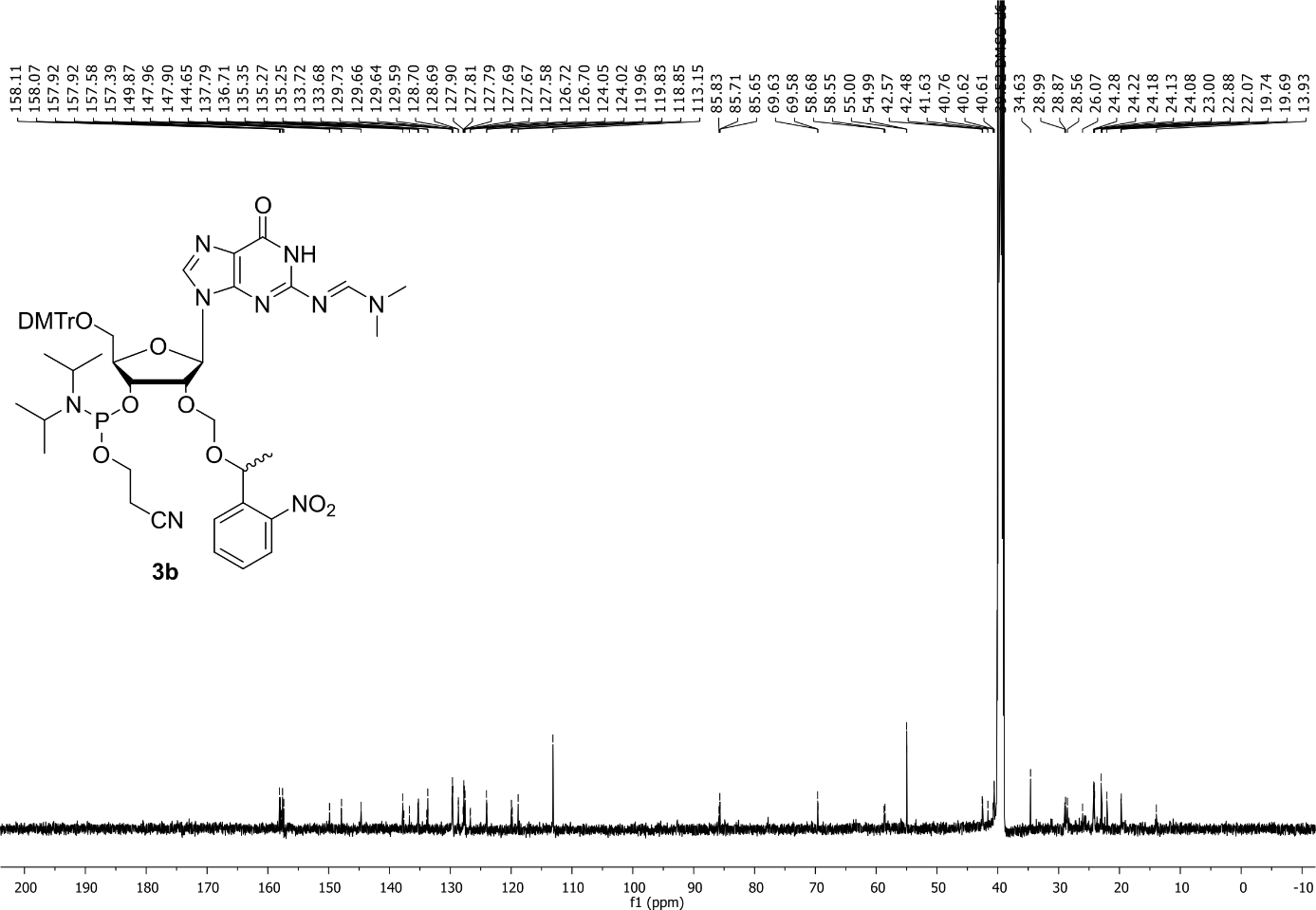


**^31^P{^1^H} (DMSO-*d*_6_):**


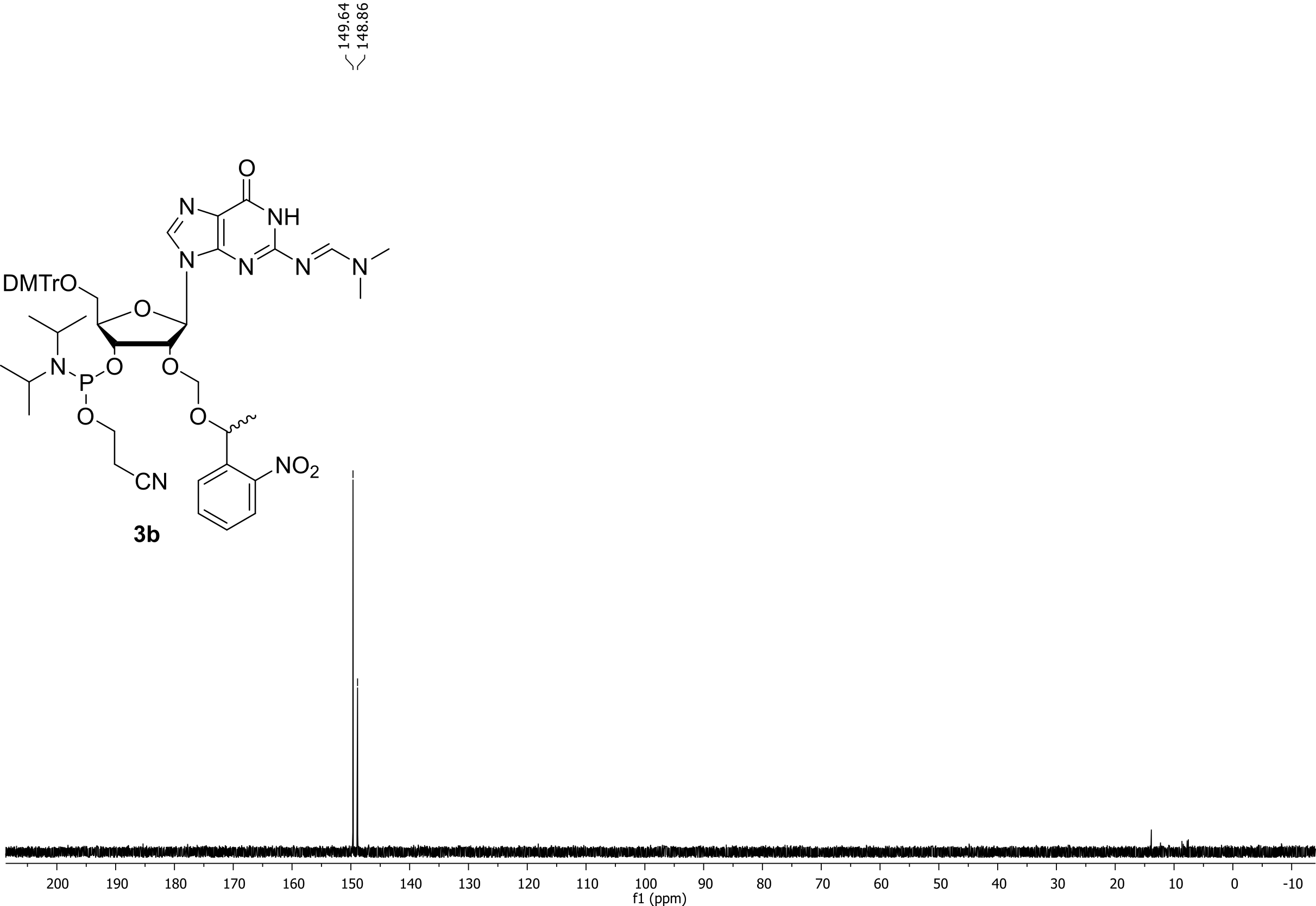


**N^4^-Acetyl-5′-O-[bis(4-methoxyphenyl)phenylmethyl]-2′-O-[[(R,S)-1-(2-nitrophenyl)ethoxy]methyl]cytidine (2c)**

**^1^H (DMSO-d_6_):**


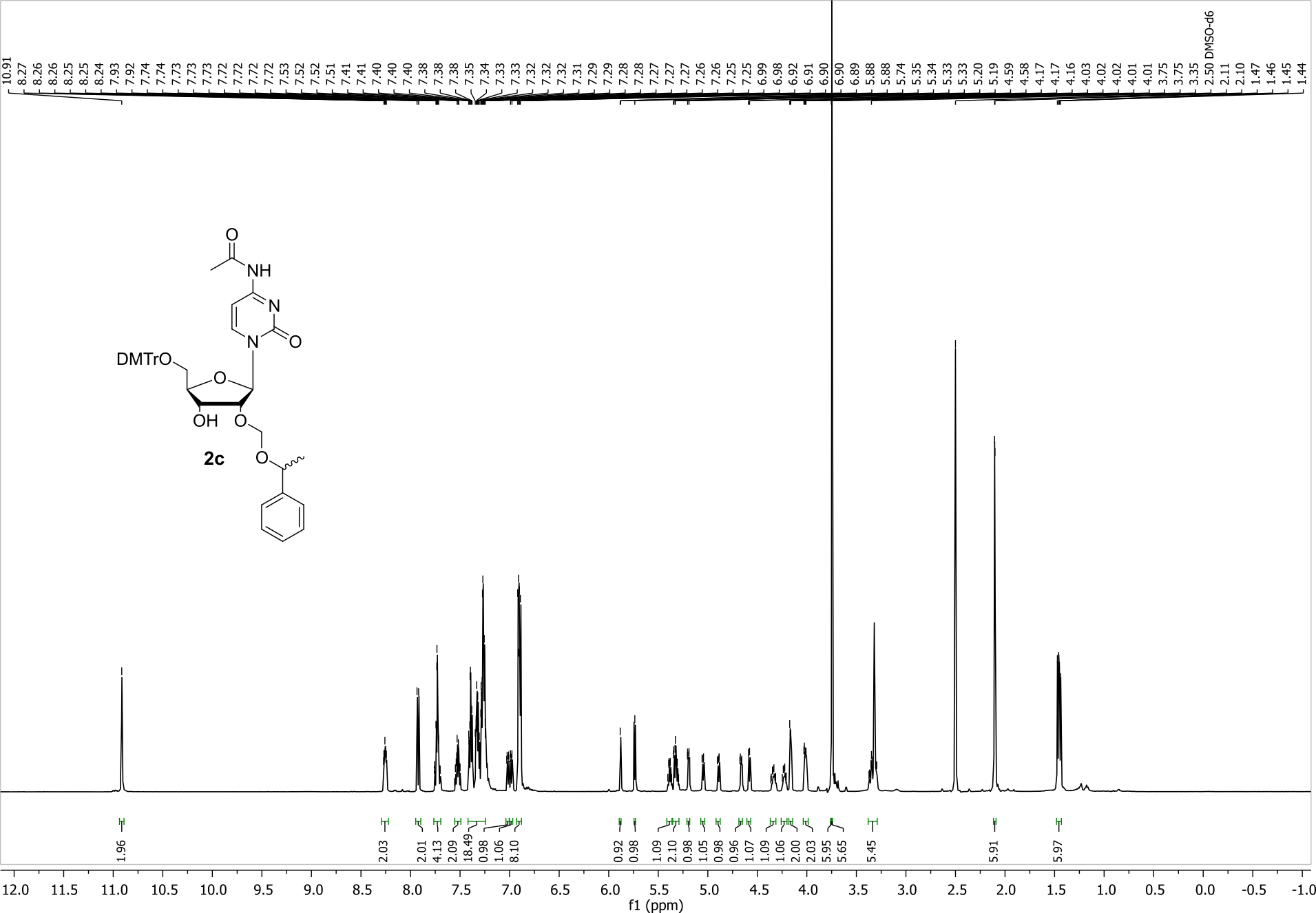


**^13^C{^1^H} (DMSO-*d*_6_):**


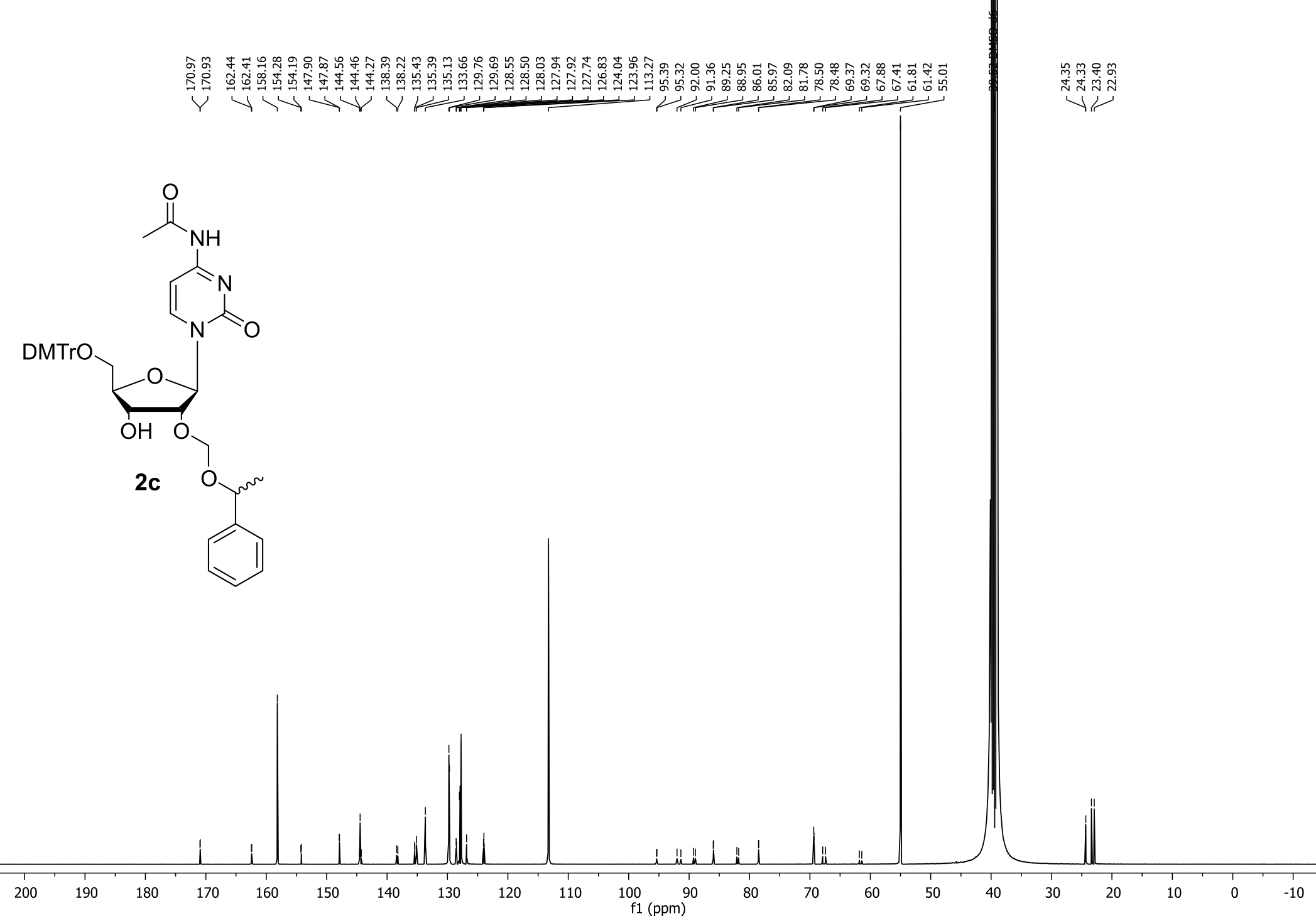


**N^4^-Acetyl-5′-O-[bis(4-methoxyphenyl)phenylmethyl]-2′-O-[[(R,S)-1-(2-nitrophenyl)ethoxy]methyl]cytidine-(2-cyanoethyl)diisopropylphosphoramidite (3c)**

**^1^H (DMSO-*d*_6_):**


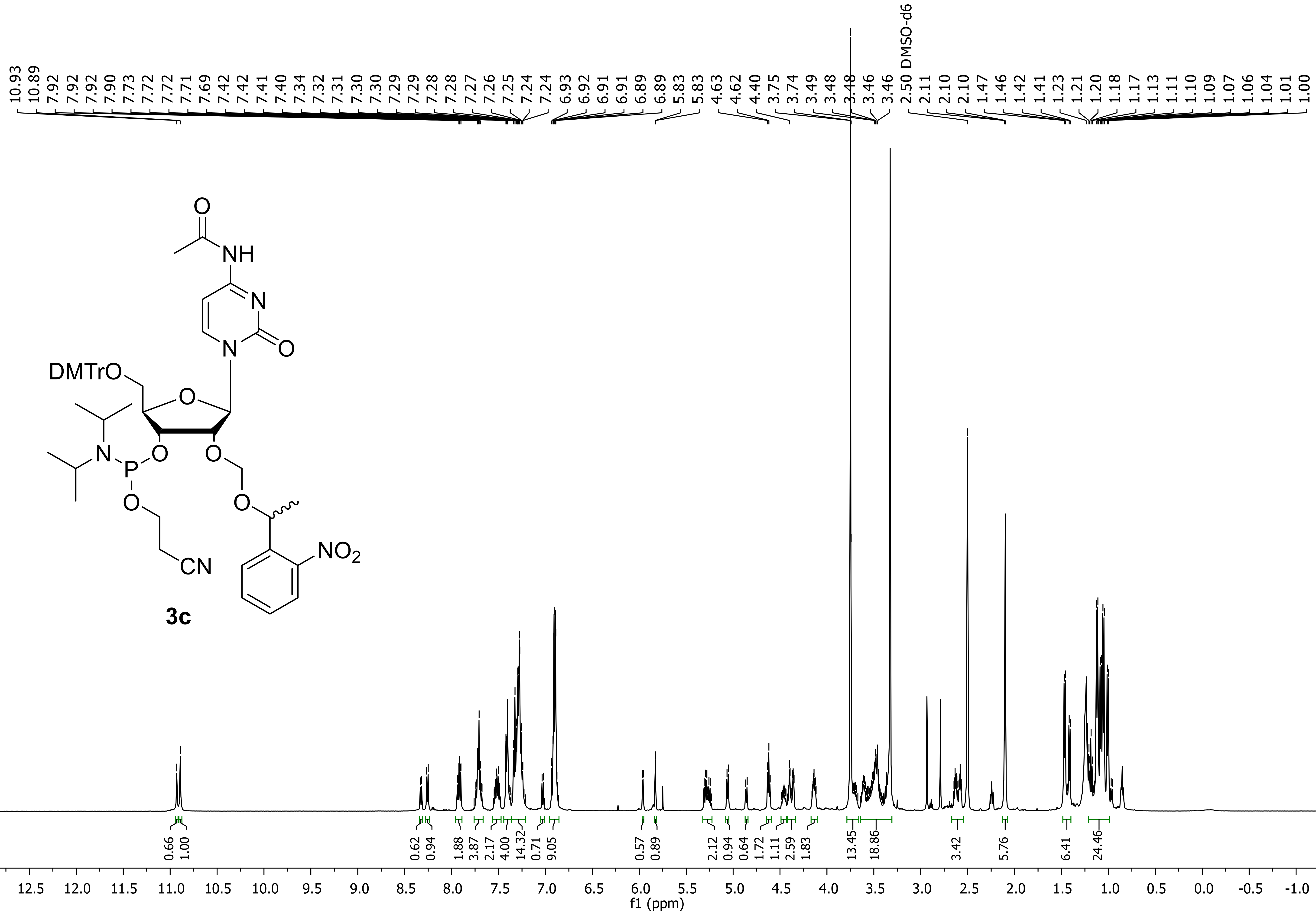


**^13^C{^1^H} (DMSO-*d*_6_):**


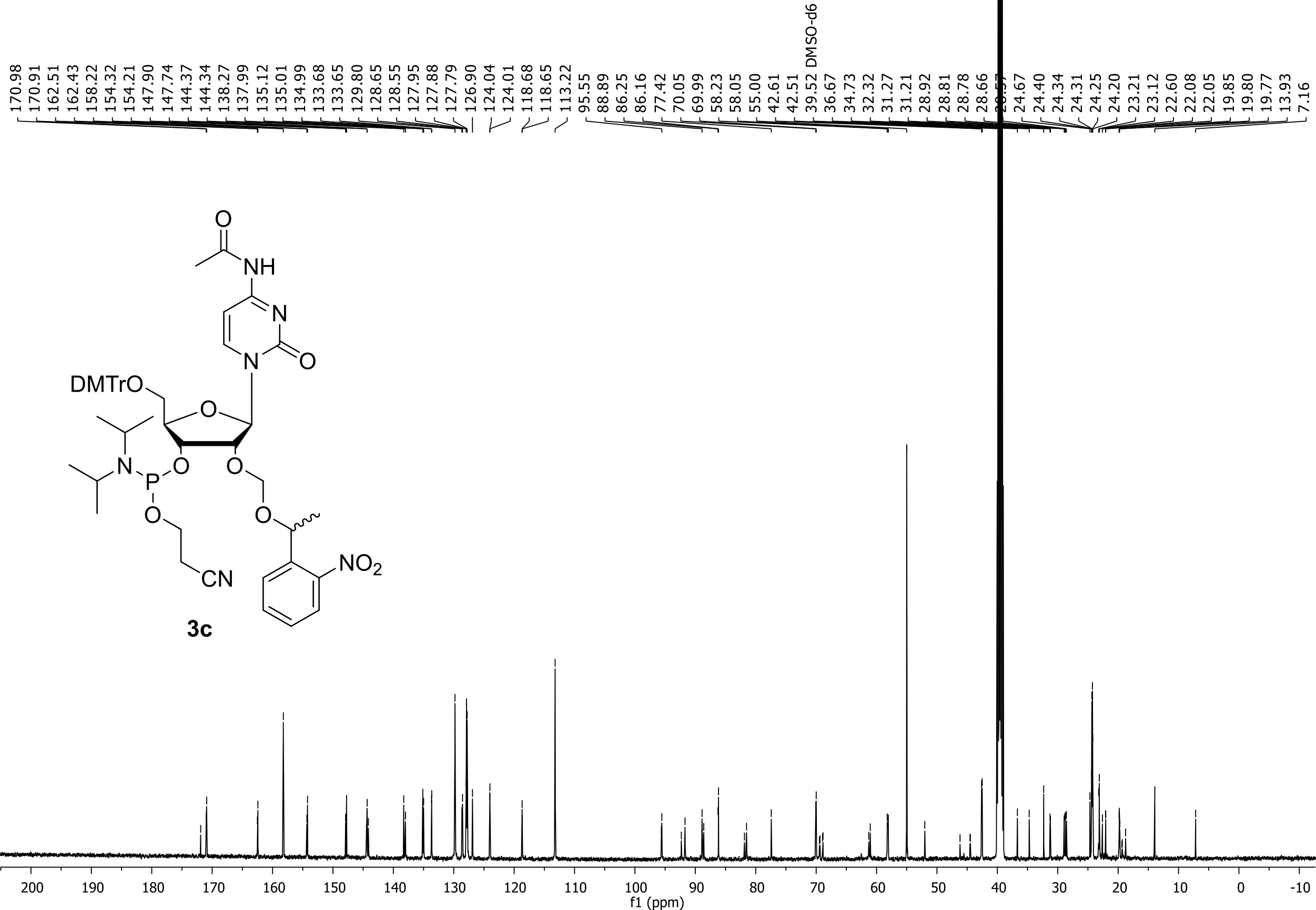


**^31^P{^1^H} (DMSO-*d*_6_):**


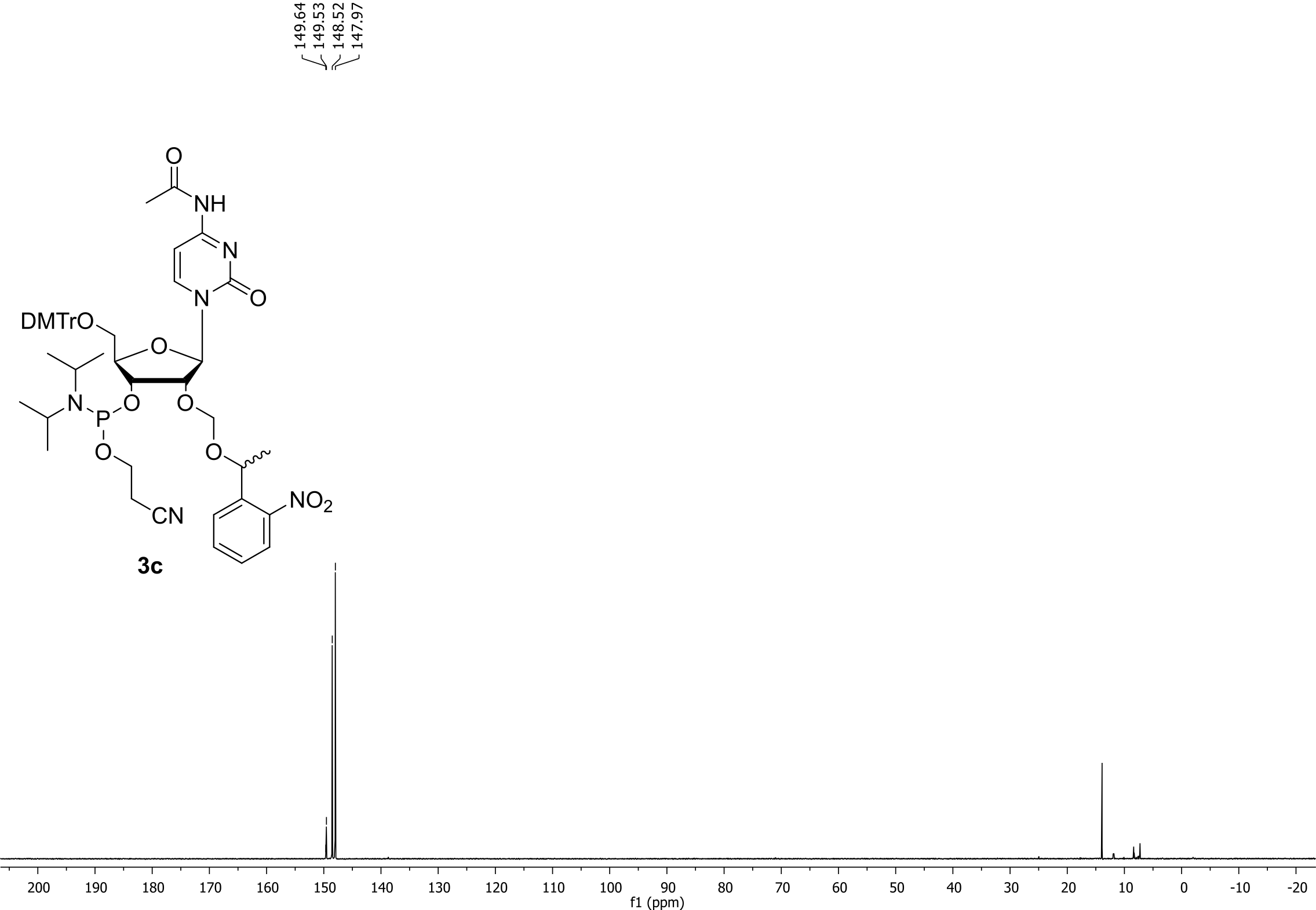


5. ESI-HRMS mass-Spectra

***N^6^*-Benzoyl-5’-*O*-[bis(4-methoxyphenyl)phenylmethyl]-2′-O-[[(R,S)-1-(2-nitrophenyl)ethoxy]methyl**]**adenosine (2a)**


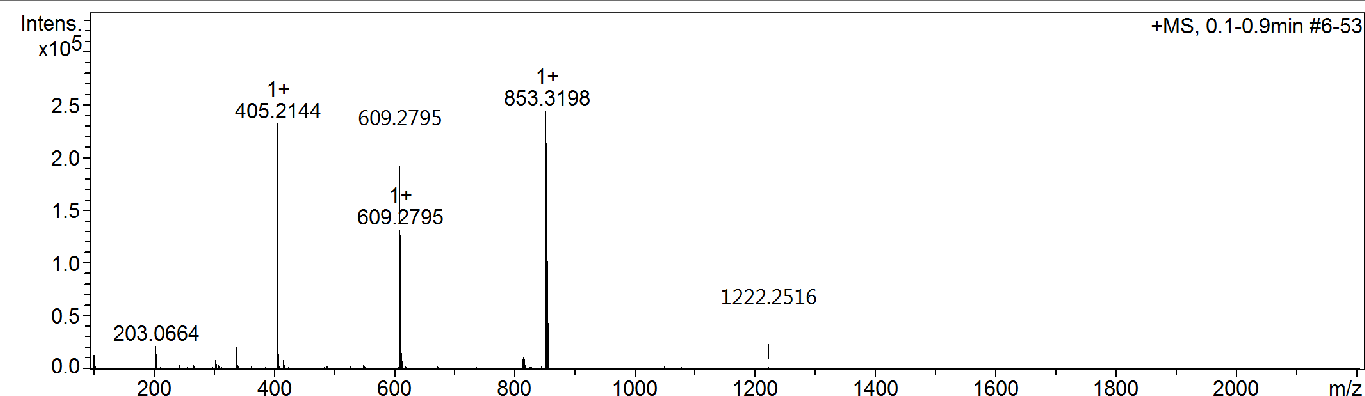


***N^6^*-Benzoyl-5’-*O*-[bis(4-methoxyphenyl)phenylmethyl]-2′-O-[[(R,S)-1-(2-nitrophenyl)ethoxy]methyl]adenosine-(2-cyanoethyl)diisopropylphosphoramidite (3a)**


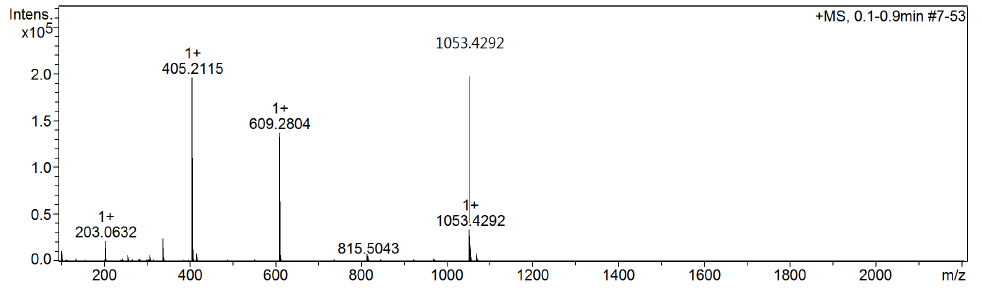


**5′-O-[Bis(4-methoxyphenyl)phenylmethyl]-N^2^-[(dimethylamino)methylene]-2′-O-[[(R,S)-1-(2-nitrophenyl)ethoxy]methyl]guanosine (2b)**


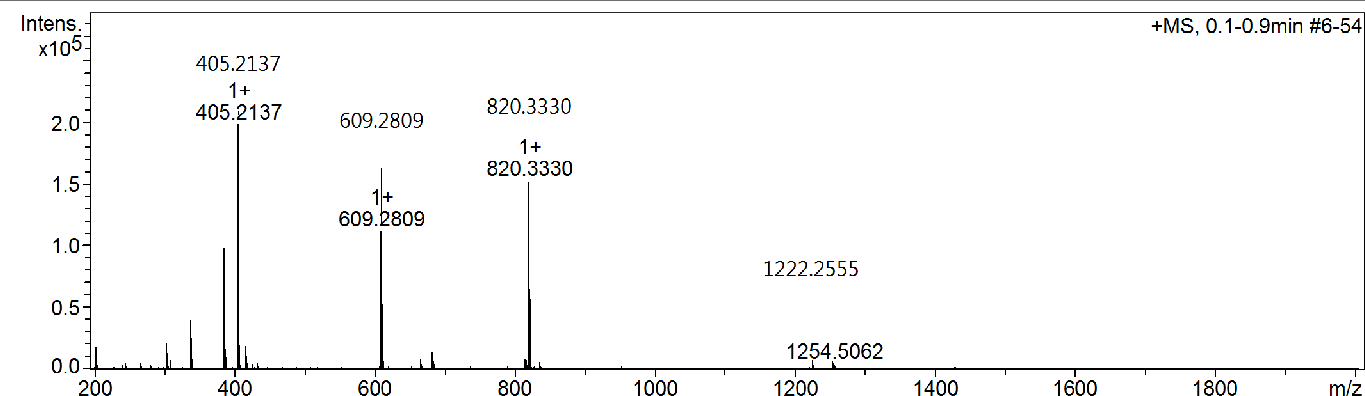


**5′-O-[Bis(4-methoxyphenyl)phenylmethyl]-N^2^-[(dimethylamino)methylene]-2′-O-[[(R,S)-1-(2-nitrophenyl)ethoxy]methyl]guanosine-(2-cyanoethyl)diisopropylphosphoramidite (3b)**


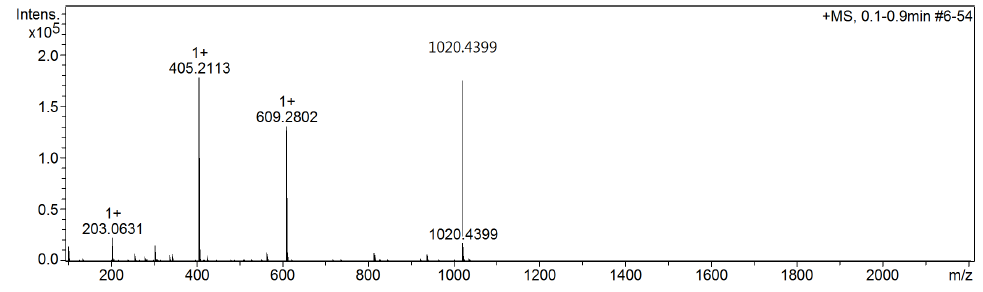


**N^4^-Acetyl-5′-O-[bis(4-methoxyphenyl)phenylmethyl]-2′-O-[[(R,S)-1-(2-nitrophenyl)ethoxy]methyl]cytidine (2c)**


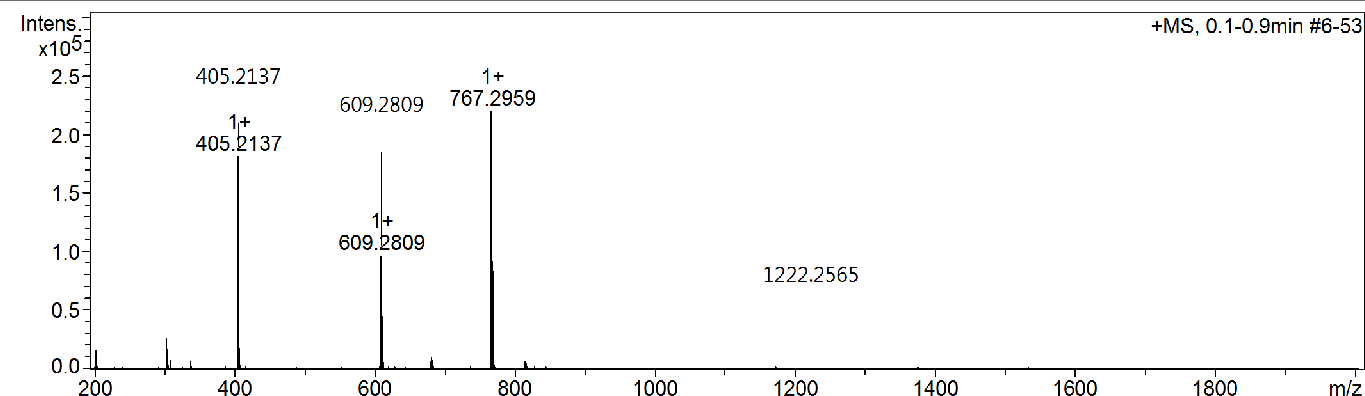


**N^4^-Acetyl-5′-O-[bis(4-methoxyphenyl)phenylmethyl]-2′-O-[[(R,S)-1-(2-nitrophenyl)ethoxy]methyl]cytidine-(2-cyanoethyl)diisopropylphosphoramidite (3c)**


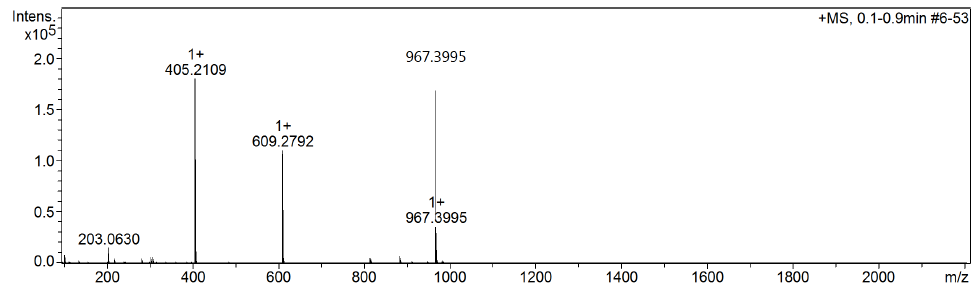


# 6. References

[1] S. Pitsch, P. Weiss, X. Wu, D. Ackermann, T. Honegger, *Helv. Chim. Acta.* **1999**, *82*, 1753-1761.

[2] M. Goldeck, T. Tuschl, G. Hartmann, J. Ludwig, *Angew. Chem. Int. Ed.*  **2014**, *53*, 4694–4698.

[3] A. G. Simon, L. K. Esser, J. Ellinger, V. Branchi, Y. Tolkach, S. Müller, M. Ritter, G. Kristiansen, M. H. Muders, T. Mayr, M. I. Toma, *J. Cancer Res. Clin. Oncol.* **2020**, *146*, 2255–2265.

# 7. Author Contributions

S.A.L. and V.R.M. contributed equally to the project. S.A.L contributed all biological experiments and analysis thereof and writing of the original draft. V.R.M contributed with chemical synthesis of all phosphoramidites and oligonucleotides and chemical analysis, as well as writing of the original draft. D.R. contributed with synthesis of the triphosphate moiety and low-resolution mass spectrometry. C.W. and T.Z. contributed equally with project administration. R.H. and M.I.T. provided primary ccRCC cells. J.L. and M.S. contributed with consultation. A.H. and G.H. initiated and conceptualized the project, and contributed funding. GH wrote large parts of the manuscript. E.B. helped with the experimental design. E.B., J.L., M.S. and T.Z. contributed to writing.
